# Do cognitive abilities reduce eyewitness susceptibility to the misinformation effect? A systematic review

**DOI:** 10.3758/s13423-024-02512-5

**Published:** 2024-05-02

**Authors:** Maryanne Brassil, Cian O’Mahony, Ciara M. Greene

**Affiliations:** 1 School of Psychology, University College Dublin, Dublin, Ireland; 2 School of Applied Psychology, University College Cork, Cork, Ireland

**Keywords:** Misinformation, Eyewitness memory, Individual differences, Cognitive abilities

## Abstract

The fact that memories can be distorted by post-event misinformation has cast considerable doubt over the dependability of eyewitness evidence in legal contexts. However, despite its adverse practical implications, the misinformation effect is likely an unavoidable distortion stemming from the reconstructive nature of episodic memory. Certain cognitive abilities have been reported to offer protection against misinformation, suggesting that mechanisms aside from episodic memory may also be underpinning this type of memory distortion. The purpose of this review was to collate findings of associations between eyewitness misinformation susceptibility and individual differences in cognitive ability in adults aged 18 and over. Nine studies met the eligibility criteria for this review, including 23 distinct associations. Using a narrative synthesis, three categories of cognitive ability were identified as influencing susceptibility to misinformation: general intelligence and reasoning, perceptual abilities, and memory abilities. Across almost all categories, higher levels of ability were associated with reduced susceptibility to misinformation. While there is no indication that any one trait provides total immunity to the misinformation effect, there is a reasonable amount of evidence to suggest that several cognitive abilities create variance in individual levels of susceptibility. Future research should investigate not only if but *how* these cognitive abilities protect against misinformation distortions, for example, by contributing to more detailed encoding of the memory, enhancing discrepancy detection in the face of post-event misinformation, or improving source-monitoring during an eyewitness memory test.

**Supplementary Information:**

The online version contains supplementary material available at 10.3758/s13423-024-02512-5.

## Introduction

It is widely understood that our memories are vulnerable to distortion and do not always accurately reflect the details of past events (Schacter, 1999). An example of one such distortion is the *misinformation effect*, whereby following exposure to post-event misinformation, incorrect details are remembered instead of the details from the original event (Loftus, 2005). The misinformation effect should be a cause for concern in any circumstance where memories are treated as a reliable source, the most notorious being the handling of eyewitness evidence during criminal justice proceedings (Benton et al., 2006; Wells et al., 2006). The misinformation effect has been studied extensively within this context (Frenda et al., 2011), with a Google Scholar search for “misinformation” and “eyewitness” yielding over 16,000 results as of May 2023. However, despite extensive research on the topic, there are still many outstanding questions regarding the exact nature of the cognitive mechanisms underpinning the misinformation effect. Furthermore, there is no definitive answer as to whether certain individual differences can provide increased protection against misinformation distortions to eyewitness memory, or whether we are all equally vulnerable.

While a hindrance to the credibility of eyewitness evidence in applied contexts, some argue that the misinformation effect is likely a side effect of typical mnemonic function, as memory has evolved to be a flexible and adaptive mechanism (Schacter, 2021; Whitehead & Marsh, 2022), allowing for reactivation and updating after encountering information (Schacter et al., 2011). However, there still appears to be variability in individual levels of susceptibility to misinformation, wherein some people may be less vulnerable to this effect than others. Individual differences in a range of cognitive abilities have been associated with reduced rates of eyewitness misinformation susceptibility, with each cognitive function having distinct effects (e.g., Greene et al., 2020; Jaschinski & Wentura, 2002; Zhu et al., 2010). This suggests that the mechanisms involved in incorporating misinformation into existing memories are more intricate, going beyond standard episodic memory functioning and involving other cognitive functions, such as short-term memory, working memory, perceptual abilities, and reasoning ability. The purpose of this review is to collate findings that have investigated the role of individual differences in cognitive ability in eyewitness misinformation susceptibility in adults to better understand their role in this type of memory distortion.

### Theoretical approaches to the misinformation effect

There are many types of false memories and memory distortions. False memories can exist in the form of recalling entirely fabricated events (Loftus & Pickrell, 1995; Murphy et al., 2023), reporting having seen a non-presented but semantically related word in a list (Deese, 1959; Roediger & McDermott, 1995) or remembering a piece of post-event misinformation rather than the original memory detail in a witnessed event (Loftus, 2005). While all these forms of false memory are thought to occur as a consequence of the reconstructive nature of memory, the false memory phenomena listed above are very weakly correlated, and it is thought that there are independent underlying mechanisms unique to each of these effects (Bernstein et al., 2018; Ost et al., 2013). Thus, this review is explicitly focused on the misinformation effect in episodic memory, to better understand the cognitive abilities associated with this specific distortion.

In an experimental context, the misinformation effect is typically studied using a three-stage paradigm. This paradigm involves witnessing an event, exposure to post-event misinformation about some details of the event, and finally recollection of the original event (Loftus, 2005). Different factors at each stage of this paradigm may impact rates of misinformation distortions. For example, certain factors at the time of witnessing the stimuli may lead to increased rates of misinformation distortion, such as the scene imposing high levels of visual perceptual load (Greene et al., 2021; Murphy & Greene, 2016) or situations where the eyewitnesses’ attention is divided during the witnessed event (Lane, 2006; Zaragoza & Lane, 1998). Typically, rates of misinformation acceptance increase in situations where cognitive resources, such as attention and executive functioning, may be put under strain, preventing the memory of the event from being fully encoded. Such findings fit well with the theory that misinformation effects often occur without actually altering any original memory detail. Instead, some researchers have suggested that post-event misinformation just fills in details that were never encoded in the first instance (Gordon & Shapiro, 2012; McCloskey & Zaragoza, 1985). In such contexts where lack of detail of the original memory encoding is the underlying cause of the memory distortion, it would follow that individuals who possess cognitive and perceptual abilities that lead to more detailed recollections would be less susceptible to misinformation overall (Greene et al., 2020). We will explore whether this assertion is supported through the studies in this review.

In a similar vein, Fuzzy-Trace Theory also posits that “gist memories” are also more prone to suggestion and distortion than more detailed “verbatim” memories (Brainerd & Reyna, 1998; Reyna et al., 2016). When encoding an episodic memory, Fuzzy-Trace Theory proposes that we simultaneously encode two representations of an event. The verbatim trace stores the details of an event as they physically appeared, whereas the gist trace stores our interpretations of the most fundamental aspects of these details. For example, a verbatim memory may capture that a thief was carrying a screwdriver in his hand, whereas the gist representation of this would be that the thief was carrying a tool with him. Gist memories are considered to be more malleable than verbatim memories. This is because both the correct detail and false details are consistent with a gist memory (e.g., remembering that the thief had some sort of tool can be consistent with remembering that the tool was a spanner instead of a screwdriver; Brainerd & Reyna, 2019). A range of factors can influence whether a gist or verbatim memory detail is recalled, including recollection cues such as the precise wording of the questions asked by an interviewer (Lampinen et al., 2006). There also is some individual variance in whether someone is more likely to recall a verbatim detail over a gist detail, and this could explain why there is some variance in susceptibility to the misinformation effect, as well as other types of false memory distortions (Brainerd & Reyna, 2019).

There are theories that provide alternative explanations to the dual-representation model of episodic memory posited by Fuzzy-Trace Theory and expand on how misinformation distortions may occur so frequently and so easily. The Source-Monitoring Framework (Johnson, 1997; Mitchell & Johnson, 2009) suggests that memory distortions like the misinformation effect occur when we confuse the source of information and misattribute the memory of a misinformation detail to the actual witnessed event. For instance, a participant might read a description of an event in which a thief carried a screwdriver (rather than a spanner), and incorrectly remember having seen the screwdriver during the original event. Strategies that boost source monitoring performance, for example, by drawing attention to the presence of misinformation in some way at the time of exposure (Bailey et al., 2021; Tousignant et al., 1986) or at the time of the memory test (Blank & Launey, 2014; Echterhoff et al., 2005), have been found to reduce misinformation effects by increasing the likelihood that eyewitnesses will notice a discrepancy between the post-event information and the original memory source. We therefore might expect that individual differences that reflect improved source-monitoring abilities will in turn reduce susceptibility to the misinformation effect in eyewitness scenarios.

### Individual differences in eyewitness misinformation susceptibility

Despite the misinformation effect in eyewitness scenarios being a widely researched phenomenon, with empirical studies spanning decades (Laney & Loftus 2018), there remains a lack of clarity over *who* exactly is vulnerable to this type of distortion. Are we all vulnerable at any given time, or are some people less susceptible than others? If so, what gives them this protection? There is no definitive answer to any of these questions to date, though tentatively we might say that the evidence points to the misinformation effect being a universal phenomenon (Patihis, 2018). For example, even those with exceptional memory ability, who can recall details of their own lives with astounding detail and accuracy, were found to be susceptible to the misinformation effect, along with other false memory distortions (Patihis et al., 2013). This finding regarding those individuals with highly superior autobiographical memories (HSAMs) highlights the inherent vulnerability of our episodic memory mechanisms to distortion and source-monitoring errors. No matter how detailed or deeply encoded the original memory may be, misinformation distortions are always possible (Schacter, 1999).

However, a caveat regarding this finding is that the ability possessed by this group of individuals appears to be limited to events about their own lives (Parker et al., 2006), so it’s unclear whether this ability would provide additional protection in cases where the memory came from being a spectator or eyewitness to an event that they were not directly involved in. Additionally, it is possible that this memory “superpower” may not actually be reflective of typical mnemonic functioning in the general population. As there are so few HSAM individuals, it is hard to know exactly what underpins their ability, but one MRI study identified structural neurological differences in HSAM in regions associated with autobiographical memory function (LePort et al., 2012). Thus, their memory retention abilities could be due to an abnormality or uniqueness in their autobiographical memory mechanisms, rather than them representing a group of individuals at the top-end of a spectrum of typical autobiographical memory function. While the fact that HSAMs are vulnerable to misinformation effects is an interesting finding, we can only cautiously extrapolate how their strengths and vulnerabilities generalise to the rest of the population.

Certain individual differences have been linked with varying levels of misinformation susceptibility in eyewitness scenarios. For example, measures of experiential traits such as fantasy-proneness and dissociative tendencies (Hyman & Billings, 1998; Porter et al., 2000) have been associated with susceptibility to misinformation and memory distortions. However, these findings show weak associations and often cannot be replicated across multiple different eyewitness misinformation paradigms (Patihis, 2018). Another individual difference creating variance in misinformation susceptibility is age, with a meta-analysis finding that older adults aged over 65 years were significantly more prone to the misinformation effect than younger adults with a moderate effect size (Umanath et al., 2019; Wylie et al., 2014). There are a myriad of reasons as to why age may influence misinformation proneness, but the mechanism with the most support relates to the decline of cognitive functioning with age (Davis & Loftus, 2005). Further supporting this point, heightened susceptibility to misinformation in older adults has been attributed to diminished source-monitoring at the time of a memory test (Bulevich & Thomas, 2012), and older adults who fare poorly on psychological tests evaluating frontal-lobe functioning demonstrate even greater challenges in source monitoring and show increased susceptibility to misinformation (Meade et al., 2012; Roediger & Geraci, 2007). These findings imply a potential connection between cognitive functioning and the observed age effects on misinformation susceptibility, highlighting the potentially crucial role of certain cognitive factors in misinformation distortions to memory.

Extensive research has also been conducted investigating the role of cognitive individual differences for children and adolescents, providing further insight into the role of cognitive functioning in this phenomenon during early stages of development. Many psychosocial factors have been associated with variance in suggestibility amongst children, with certain studies finding that cognitive abilities are linked to memory distortions such as the misinformation effect (for comprehensive reviews on the topic, see Bruck & Melnyk, 2004; Klemfuss & Olaguez, 2020). However, as these reviews highlight, while performance on intelligence, executive functioning, and working memory tasks sometimes predict susceptibility to misinformation effects in children, this is not always replicated, with many studies not finding any link between cognitive abilities and the misinformation effect. Klemfuss and Olaguez (2020) note in their update of this review that the reason for these inconsistent findings surrounding misinformation effects and cognitive ability in children is still not clear.

These reviews on suggestibility in children also highlight a range of other individual difference factors that influence vulnerability to misinformation and leading questions in eyewitness paradigms. For example, developmental differences in language comprehension and production are quite consistent predictors of suggestibility in children (e.g., Curci et al., 2017; Klemfuss, 2015). Furthermore, understandings of the mechanisms underpinning eyewitness suggestibility in children are further clouded by the fact that while children were thought always to be more susceptible to misinformation and leading questions compared to adults (Knutsson & Allwood, 2014), some studies have identified that children’s lack of existing knowledge and schematic representations of events can, in some cases, render them less susceptible to memory distortion compared to adults, as they are less likely to incorporate information to fit their expectations of certain events (Otgaar et al., 2018).

While understanding the contributions of individual differences in cognitive ability for misinformation susceptibility in eyewitnesses of all ages is crucial, we are excluding studies with children and adolescents from this review for two reasons. The first is that the reviews conducted by Bruck and Melnyk (2004) and Klemfuss and Olaguez (2020) on individual differences in suggestibility in children included studies that looked at cognitive factors in misinformation effect paradigms, so there are existing reviews that comprehensively cover this research question in populations under the age of 18 years. The second and most pertinent reason for exclusively focusing on adult populations in our review is that pronounced developmental differences in cognitive ability would make direct comparisons between children and adults challenging, and that notable differences in language skills and knowledge bases of children, especially younger children, could confound results, making it difficult to isolate the exact cognitive mechanisms underpinning eyewitness misinformation effects.

We argue that the subset of individual differences relating to specific cognitive functions may reveal a lot about the underlying mechanisms of the misinformation effect in adults. Cognitive abilities are typically relatively stable traits within individuals that allow for and benefit the execution of several tasks in day-to-day life (Carroll, 1993). For example, working memory allows for the simultaneous storage of information, and processing of information for problem-solving (Daneman & Carpenter, 1980). Working memory capacity varies from person to person and is critical to an array of tasks and problem-solving in everyday life, from following a recipe when cooking to having a conversation with a friend (Richmond et al., 2015), or distinguishing between relevant and irrelevant information when recalling information (Lilienthal et al., 2015; Unsworth & Brewer, 2010). Thus, it is plausible that certain cognitive abilities, such as working memory capacity, could contribute to resisting misinformation effects in eyewitness contexts (and previous associations have been found; Jaschinski & Wentura, 2002). A number of other cognitive abilities have been associated with a reduction in eyewitness misinformation susceptibility; for example, Zhu et al. (2010) identified significant associations with measures of intelligence tasks, perceptual ability, and short-term memory. However, all of the research on the link between cognitive abilities and eyewitness misinformation susceptibility in adults has not yet been collated and examined regarding what it reveals as a whole.

### The current review

This review aims to systematically synthesize findings from studies investigating the relationship between individual differences in cognitive ability and rates of the misinformation effect in an eyewitness memory paradigm. Despite decades of research into the misinformation effect and its underlying mechanisms, to our knowledge, this is the first review with a question that focuses on the contributions of cognitive abilities to eyewitness misinformation susceptibility in adults. While many commentaries and papers state that cognitive ability does impact eyewitness misinformation susceptibility to a certain extent (Frenda et al., 2011; Laney & Loftus, 2018), there is no existing resource that has summarised and examined all published literature on this topic to determine whether there is sufficient evidence to reach this conclusion. The goal of this review is to provide a clear overview of the state of the literature on this topic, to identify exactly which cognitive abilities have been linked with eyewitness misinformation susceptibility, to discuss its current implications for the debate over whether all people are equally susceptible to misinformation distortions to memory, and to identify gaps in the literature that require further investigation.

## Methods

### Identifying relevant articles

#### Protocol

The protocol for this review was created using PRISMA-P guidelines (Moher et al., 2015). It was registered on PROSPERO on 23.04.2021 before conducting searches (https://www.crd.york.ac.uk/prospero/display_record.php?RecordID=250947). The protocol was updated on 21 May 2021 to clarify certain eligibility criteria before full-text review.

#### Eligibility criteria

For inclusion in this review, articles needed to be published in a peer-reviewed journal with an English language version available. No publication date limitations were set. Further eligibility criteria for study characteristics were specified using the headings from the PICO model (Richardson et al., 1995).

*Population:* The study population must have consisted of adult humans. Studies were excluded if their sample consisted entirely of a clinical population with a specific developmental, cognitive, or mental health condition. Studies with children were also excluded.

*Interventions:* Studies must have implemented an eyewitness misinformation paradigm, with three necessary stages. Firstly, a stimulus must have been presented to create a novel episodic memory in participants: for example, a video clip, audio clip, picture slideshow, or live event. This must have been followed by exposure to post-event misinformation about details of the witnessed event, and finally, a measurement of accuracy on items for which misinformation was presented. Studies that only elicited false memories using other types of paradigms (e.g., Deese-Roediger-McDermott word-list paradigms, and autobiographical false memory paradigms) were excluded, as it is believed that the cognitive mechanisms underlying these different types of memory distortions are distinct from those associated with the misinformation effect (Falzarano & Siedlecki, 2019). Eyewitness line-up studies were also not included. Finally, studies in which participants are asked to “self-generate” misinformation or lie about the events they witnessed were not included.

*Comparator:* Studies that did not have a valid experimental control for the post-event misinformation items were excluded. This control could either be between subjects or within subjects, as long as a memory for post-event misinformation items could be compared with post-event control items.

*Outcome:* Findings must be a measure of cognitive ability and a measure of memory accuracy for details on which misinformation was provided. These measures of cognitive ability were required to be performance-based behavioural measures. Studies where cognitive ability was only measured using self-report or observational measures were excluded. Measures of personality or experiential traits were excluded, as were ratings of confidence in one’s memory ability or similar meta-cognitive judgements.

#### Search strategy

Four electronic databases (Academic Search Complete, PsycINFO, Scopus, Web of Science) were searched using keywords and MeSH terms relating to the categories of Eyewitness, Misinformation, and Cognitive Ability. To minimise the risk of missing potentially eligible articles, no filters were used on any of the databases relating to article type or language. The exact search terms were finalised during a consultation with a university librarian to ensure that all searches were appropriate for each database. The search strings used in each of the four databases are available in the Online Supplemental Materials (OSM). Following full-text review, the reference lists, and if available, lists of articles that have since cited the included article, were screened to identify additional potentially eligible articles for full-text screening. The final search was performed on 16 January 2023.

#### Screening

All records were uploaded to Endnote (2013), where duplicates were removed using instructions from Bramer et al. (2016). The remaining articles were then uploaded to Covidence (2021) for the title and abstract screening. All titles and abstracts were screened by two independent reviewers (MB and CO), with any conflicts being resolved by a third reviewer (CG). Double screening of full texts was then conducted by the same two reviewers, with the third reviewer resolving any conflicts once more. Following the completion of full-text screening, the reference lists, and if available cited-by lists, for eligible articles were scanned for additional references. Any potentially eligible additional references were uploaded to Covidence where they were again screened against the eligibility criteria by two reviewers.

Interrater reliability using Cohen’s Kappa was fair (k = 0.30) for the title and abstract screening. Reviewers discussed the discrepancies and potential issues with clarity in the eligibility criteria, after which the review protocol was updated on 21.06.21 to clarify that studies that ask participants to self-generate misinformation or lie were to be excluded and that studies that only included measures of meta-memory judgements and measures of self-reported proneness to cognitive distortions, rather than performance in a cognitive task, were also to be excluded. Following this amendment, the kappa for inter-rater agreement increased to moderate (k = 0.55) for full-text review. The exact number of agreements and disagreements during the screening process are available in the OSM.

### Data management

#### Data extraction

The following items of information were extracted from each eligible study: (1) Publication information, (2) research aims and hypotheses, (3) participant information (inclusion and exclusion criteria, sample size, sampling strategy, demographic information), (3) eyewitness misinformation paradigm (descriptions of eyewitness stimuli, misinformation and control items, procedure, coding of responses, measurement of the misinformation), (4) relevant results (description of cognitive ability measure(s), descriptive statistics, inferential statistics for associations between cognitive ability measure(s)) and eyewitness memory performance (test value, *p* values, effect sizes, confidence intervals), any additional analysis (e.g., covariates, missing data management), and authors’ interpretation of findings. Studies where statistical results and effect sizes were not reported and could not be extracted, were excluded from the synthesis.

#### Quality appraisals

All results from the eligible studies were individually assessed for methodological and reporting quality using the Cochrane Risk of Bias 2 tool for individually randomised parallel-group trials. Each reported association between a cognitive function and misinformation susceptibility included in the synthesis was rated as posing a low risk of bias, high risk of bias, or some concerns regarding bias in five domains: (1) The randomization process, (2) intended interventions, (3) missing outcome data, (4) measurement of the outcome, (5) selection of the reported result. Based on the judgement in the five domains, an overall risk of bias assessment was provided for each outcome variable. The ROB2 was selected as this tool allows for the assessment of quality and bias specific to the included result, rather than assessing for bias in the study overall, which may have included other findings not relevant to the review question (Sterne et al, 2019). Risk of bias appraisals were conducted by the first author (MB), using the published guidance for the RoB2 tool, and the accompanying template on Microsoft Word.

#### Synthesis approach

Study characteristics, such as research design, misinformation intervention, measurement of misinformation effect, and outcome measures were tabulated to see if the findings from included studies were suitable for statistical meta-analysis. However, given the small number of included studies, and significant variation in the measurement of the misinformation effect across included studies, it was determined that results were not suitable for meta-analysis. The heterogeneity of measurement in misinformation effect studies has been noted by other earlier systematic reviews, which have also utilised narrative syntheses to collate findings on associations between susceptibility to misinformation and emotion (Sharma et al., 2022) and to review how false memories and memory distortions are induced in laboratory settings (Muschalla & Schönborn, 2021). Thus, narrative synthesis was deemed the most appropriate tool to answer our review question and was conducted following the guidelines from Popay et al. (2006). Reported effect sizes and *p* values for each association between a measure of cognitive function and misinformation susceptibility are specified in the results section.

## Results

### Study selection

**Fig. 1:**
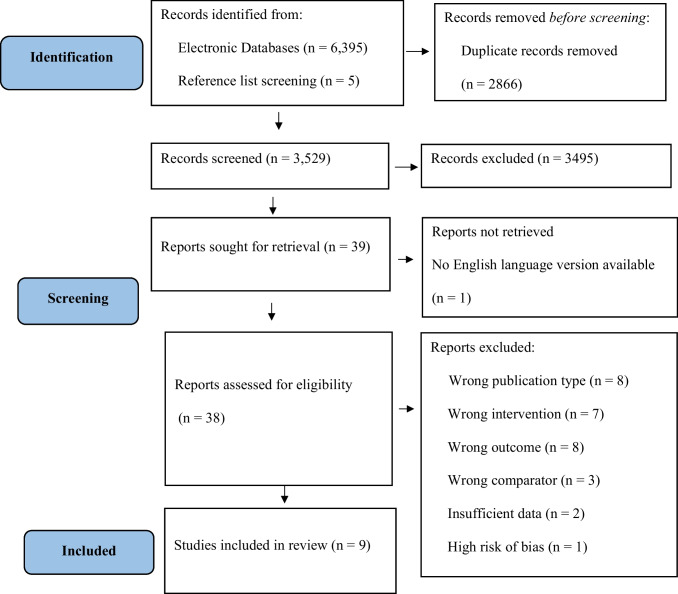
PRISMA (Preferred Reporting Items for Systematic Reviews and Meta-Analyses) flow chart of study selection process

In total, 6,395 records were retrieved from electronic database searches, with 3,529 remaining after all duplicates were removed. Following title and abstract screening, 34 articles remained for full-text review. At this stage, reference lists of all 34 articles were scanned for any other potentially relevant articles missed by the database searches. If available, lists of articles which had cited the included study since its publication were also screened. An additional five articles were identified at this stage and were added to the full-text review. It was not possible to include one article in the full-text review as no English language version of the paper was available (Guo & Li, 2007), leaving 38 articles to be screened. Following full-text screening, 12 articles were identified as having at least one eligible finding to be included in this review. However, two of these articles were excluded at the data extraction stage, as no statistical results on the association between the cognitive ability measure were made available in the paper (Eisen et al., 2013; Kiat et al., 2018). Another article was excluded during the quality appraisal stage, as all relevant findings were deemed to be at high risk of bias (Powers et al., 1979). This left a total of nine studies to be included in this review. Figure 1 outlines a PRISMA flow chart of the article selection process.

### Included studies

Nine studies were included in this review. Within these nine studies, there were 23 associations between eyewitness misinformation susceptibility and individual differences in a cognitive ability. However, it should be noted that 11 of these findings came from the same study (Zhu et al., 2010), with the other 12 from across the remaining eight studies.

### Quality appraisals of included studies

Findings that posed a high risk of bias in more than one domain were excluded from the synthesis, which in this instance was one study that included three potentially relevant findings (Powers et al., 1979). The primary concern related to the measurement of cognitive ability employed by this study. Three subscales of the Washington Pre-College Test (WPCT) were investigated with regard to their association with eyewitness misinformation susceptibility. The WPCT is a discontinued aptitude test that was administered in US high schools, similar to the SAT (Scholastic Aptitude Test; Lunneborg, 1966). This study took participants’ existing WPCT scores from their college admission records and did not administer the test in a standardised setting. Thus, the content of the test and the context in which it was administered may have been highly variable across participants, and all authors agreed that it was not suitable for this synthesis.

Aside from this excluded study, one study (Tomes & Katz, 1997) was judged as presenting “some concerns of bias” across two domains, as the article provided limited information on the randomisation process and employed substantially different sample sizes in the experimental group (n = 132) and control group (n = 48). This study was nevertheless included in the narrative synthesis. Additionally, all included studies with the exception of Greene et al. (2021) received a judgement of “some concerns” in the bias of the reported result domain, as no pre-registered analysis plan was made available. Greene et al. (2021) received a “low” concerns judgement as a pre-registered analysis plan was available. More details on the results of the quality appraisals in this study are available in the OSM.

### Description of included studies

#### Publication details

All included articles were peer-reviewed journal articles published in the English language. The publication date ranged from 1997 to 2021. Four studies were conducted in North America, four in Europe, one in China, and one in New Zealand. The participant pool in the majority of the studies consisted entirely of the student population at the university in which the principal investigator conducted the study (n = 6), with two studies being conducted with an international sample via the online recruitment platform Prolific (Farina & Greene, 2020; Greene et al., 2020), and one study being conducted online through social media advertising and snowball sampling (Greene et al., 2021). The reported average age of participants in most of the included studies was under 35 years (n = 8). One study did not report any information about participants’ ages (Parker et al., 2008). Additionally, while Tomes and Katz (1997) reported an age range between 17 and 43 years, it was ultimately deemed eligible for inclusion in this review, given the youngest participant was just 1 year younger than our specified lower age limit (18 years), and the mean age in the sample was 20 years.

#### Eyewitness misinformation paradigms

All included studies implemented an eyewitness misinformation paradigm (Loftus, 2005), consisting of the essential stages of encoding the novel eyewitness memory, misinformation exposure, and an eyewitness memory test. Most included studies used a within-subjects design, with misinformation and control items counterbalanced across participants (n = 7), as opposed to less frequent use of between-groups design (n = 2). There was much variability in the media used to present each stage of the eyewitness misinformation paradigm, and the details of the materials used in each study are outlined in Table 1.

**Table 1:** Experimental design and misinformation effect measurement of included studies, listed in chronological order

**Study**	**N**	**Participant demographics**	**Experimental context**	**Original EW stimulus**	**MI presentation format**	**Design** **(MI vs. control)**	**Memory Test**	**Delay**	**MI effect measurement**	**Sig. MI effect?**
Tomes & Katz (1997)	180	Age range = 17–43 years; Mean age = 20.00 years; Gender = 90 male, 90 female	Laboratory setting	Video clips (3 x 1 min.)	Leading questions (12 MI items)	Between groups	Open-text cued recall	15 min	Accuracy of responses for each MI item was compared to control group accuracy for the same item, and items where there was a significant difference were included in primary analysis (7/12 items) Participants were then grouped on the basis of whether or not they provided misinformation as a response for at least one item in all 3 video clips (habitually susceptible vs. not habitually susceptible groups)	Yes. MI accuracy higher than control accuracy for 4 of the included items at *p* < .05. Three more items “approached significance” (*p* < .08) and were included in primary analysis.
Jaschinski & Wentura (2002)	38	Age range = 20–38 years; Median age = 21 years; Gender = 9 male, 29 female	Laboratory setting	Video clip (1 x 10 min)	Scrambled narrative (4 MI items)	Within subjects	Open-text cued recall	Not specified (< 1 hour)	Primary analysis used total misinformation effect score (0-4), computed as control item correct responses minus misinformation item correct responses. Control item correct responses (0-4) and misinformation item correct responses (0-4) were also separately analysed in relation to the cognitive ability variable	Yes. Mean correct for control items was significantly great than mean correct for MI items.
Parker et al. (2008)	76	No demographic information provided.	Laboratory setting	Slideshow (1 x 3 min)	Chronological narrative (4 MI items)	Within subjects	Multiple choice	3 min	Misinformation item correct responses (0-4)	Yes. Mean correct responses for control items greater than mean correct for MI items.
Zhu et al. (2010)	436	Mean age = 19.72; Standard deviation = 0.94; Gender = 55% female^†^	Laboratory setting	Slideshow (2 x 3 min)	Chronological narrative (2 x 12 MI items)	Within subjects	Multiple choice	10 min	Overall FM = % misinformation items endorsed; Robust FM = % of misinformation items endorsed and reported seeing in misinformation in original event only, or both original event and narrative	Yes. MI endorsement greater than foil item endorsement for both overall FM and robust FM.
Calvillo (2014)	75	Mean age = 20.15; Gender = 16 male, 50 female	Laboratory setting	Slideshow (2 x 3 min)	Chronological narrative (2 x 12 MI items)	Within subjects	Multiple choice	Not specified (< 1 hour)	% of misinformation items endorsed	Yes. MI endorsement great than foil endorsement.
Nichols & Loftus (2019)	297	Mean age = 20.80; Gender = 89 male, 277 female, 7 unreported; Ethnicity = Asian: 42.6%, Latino: 25.1%, Caucasian/ White: 15.3%, Middle Eastern: 4.6%, African American/ Black: 1.6%, Hawaiian or Pacific Islander: 0.5%, U.S. Indian: 0.0%, Multiple ethnicities: 9.0%, Other: 1.1%	Laboratory setting	Video clip (1 x 6.5 min)	Chronological narrative (4 MI items)	Within subjects	Multiple choice	5 min	Overall FM = % misinformation items endorsed; Robust FM = % of misinformation items endorsed and reported seeing in misinformation in original event only, or both original event and narrative	Yes. Participants more accurate for control items than items where they were misled.
Farina & Greene (2020)	264	Age range = 18–70 years; Mean age = 34.39; Standard deviation =11.81; Gender = 67 male, 197 female	Online	Video clip (1 x 1 min.)	Leading questions (3 MI items)	Between groups	Leading questions (Time 1) Open-text cued recall (Time 2)	Immediate (Time 1) 7 days (Time 2)	% misinformation item accuracy	Yes. Reduced accuracy in response to leading vs. non-leading questions.
Greene et al. (2020)	219	Age range = 18–65 years; Mean age = 28.94; Standard deviation = 10.75; Gender = 100 male, 119 female	Online	Video clip (1 x 4 min)	Chronological narrative (6 MI items)	Within subjects	Open-text cued recall	5 min	% misinformation item accuracy	Not significant, but mean control item accuracy was higher than mean MI item accuracy.
Greene et al. (2021)	1165	Age range = 18–74 years; Mean age = 29.80; Standard deviation = 12.78; Gender = 297 male, 856 female, 5 other gender	Online	Video clip (1 x 2 mins)	6 different methods: Doctored photograph, chronological narrative, scrambled narrative, fill-in-the-blank narrative, elaborate leading questions, simple leading questions (All 2 MI items)	Within subjects	Half open-text cued recall and half binary choice questions (counterbalanced within groups)	Less than 15 min	Misinformation item endorsement (0–2)	Yes. More misinformation reported in MI condition than in control condition.

Study = Citation; N = Number of participants; Participant demographic = All reported demographic information for participants in each study; Test context = The context in which the experiment was administered to participants, e.g., online vs. in a laboratory setting, EW stimuli = Eyewitness stimuli medium; MI presentation = Misinformation item medium; Control = Type of experimental control; Memory type = Method through which participants were evaluated on memory of EW stimuli; EW stimuli; Delay = Delay between misinformation exposure and memory test; MI effect measurement = How significant misinformation effect was defined; Sig. = Significant difference between misinformation item scores and control items scores at p < .05 level

†In Zhu et al. (2010), demographic information was only reported for the 557 participants recruited for a larger project, not the subset of 436 included in this specific study

As was expected, the majority of studies found a significant misinformation effect, wherein memory accuracy for items on which misinformation was provided was significantly less than that for control items (n = 8). Greene et al. (2020) did not find a significant effect when comparing misinformation item accuracy versus control item accuracy in a pairwise t-test (*p* = .07), but descriptive statistics indicated that accuracy was still reduced for misinformation items compared to the control items.

#### Cognitive functions and eyewitness misinformation susceptibility

While only nine studies met the eligibility criteria for inclusion in this review, many of the included studies contained multiple associations between different measures of individual differences in cognitive ability, as measured by performance in a behavioural task. The 23 findings from these nine studies were categorised into three cognitive ability types. The first type is general intelligence and reasoning, which includes nine findings: two measures of non-verbal IQ, three measures of verbal IQ, two spatial reasoning measures, and two verbal analytical reasoning measures. The second type is perceptual abilities, which includes four findings: three in the domain of visual perception, and one measure of auditory perception. The third type is memory abilities, which includes ten findings: five measures of working memory capacity, four measures of short-term memory recall and recognition, and one measure of autobiographical memory specificity. Descriptions of all cognitive ability tasks implemented across the nine included studies are available in the supplemental materials.

Out of the 23 findings included in the synthesis, 21 of these findings found that higher levels of the cognitive ability variable significantly reduced eyewitness susceptibility to misinformation to some extent, with the two non-significant findings both falling under the category of a verbal intelligence or verbal analytical reasoning measure. Details on internal reliability estimates for eyewitness memory tests were not reported in studies, with the exception of Zhu et al. (2010), who reported substantial correlations between the two misinformation tests used (r(435) = .58, *p* < .001), with internal consistency equivalent to Cronbach’s alpha = .76. However, this was as expected, as due to the counterbalancing of items across misinformation and control conditions in experimental designs, it is often not possible to calculate reliability estimates (see Nichols & Loftus, 2019). Internal consistency, split-half reliability estimates, and test-retest reliability for the cognitive tasks are noted in Table 2 where they were reported, indicating sufficient levels of reliability in most tasks employed. The full list of cognitive functions investigated, and a summary of the main findings are listed in Table 2.

**Table 2:** Cognitive functions in included studies and main findings on link between functions & misinformation susceptibility

**Study**	**Cognitive function**	**Task**	**Task reliability**	**Group analysed**	**Stat. test**	**Results**	**Finding Description**
Tomes & Katz (1997)	Spatial imagery rotation	Paper Folding Task (Ekstrom et al., 1976)	Not reported	Only those exposed to misinformation; control group excluded (132/183 participants)	Pearson’s Correlations & Chi-square	r(57) = -.20, p < .01** χ2(1,132) = 2.48, p = 0.12	Imagery manipulation ability negatively associated with habitual MI acceptance across 3 videos. Not significant in Chi-square analysis
Tomes & Katz (1997)	Spatial imagery rotation	Card Rotation Task (Ekstrom et al., 1976)	Not reported	Only those exposed to misinformation; control group excluded (132/183 participants)	Pearson’s Correlations & Chi-square	r(57) = -.20, p < .01** Chi-square not reported. Assumed not significant as only sig. or “approaching significant” results were reported for the Chi-square analysis	Imagery rotation ability negatively associated with habitual MI acceptance across 3 videos. Not significant in Chi-square analysis
Jaschinski & Wentura (2002)	Working memory capacity (WMC)	Operation-Word Span (Turner & Engle, 1989)	This study: Cronbach’s alpha = 0.87	All participants	Pearson’s correlation	r(37) = -.35, p < .05*	WMC negatively associated with MI susceptibility
Parker et al. (2008)	Working memory capacity (WMC)	Operation Span (Unsworth et al., 2005)	Cited Unsworth et al., 2005: Test-retest reliability *r* = .83; Cronbach’s alpha = .78	Separate analyses for “placebo effect group” (told they were given cognitive boosting drug) (36/76) and “no placebo group” (told they were given inactive drug) (40/76)	Partial Pearson’s correlations for the two groups	Placebo group: r(33) = -.28, p < .05* No placebo group r(37) = .06, p > .05	WMC negatively associated with MI susceptibility in placebo group told they were given a cognitive boosting drug, but not in no placebo group
Zhu et al. (2010)	Non-verbal intelligence	Raven’s Advanced Progressive Matrices (Raven et al., 1998)	This study: Cronbach’s alpha = 0.75; Cited Zhai 1999: Split-half reliability *r* = 0.86	All participants	Pearson’s correlation	r(434) = -.35, p < .001***	General intelligence negatively associated with MI susceptibility
Zhu et al. (2010)	Non-verbal intelligence	Weschler Adult Intelligence Scale - Performance (Gong et al., 1992)	Cited Gong et al., 1992: Test-retest reliability* r* = .89; Split half reliabilities between *r =* .67 and *r =* .75	All participants	Pearson’s correlation	r(434) = -.29, p < .01**	General intelligence negatively associated with MI susceptibility
Zhu et al., 2010	Verbal intelligence	Weschler Adult Intelligence Scale – Verbal (Gong et al., 1992)	This study: Cronbach’s alpha: = .82; Cited Gong et al., 1992: Test-retest reliability *r* = .89; Split half reliabilities in specific tasks in the battery were between *r =* .58 and *r =* .89	All participants	Pearson’s correlation	r(434) = -.13, p < .01**	General intelligence negatively associated with MI susceptibility
Zhu et al., 2010	Working memory capacity	2-back Task (Xue et al., 2004)	This study: Cronbach’s alpha = .82	All participants	Pearson’s correlation	r(434) = -.17, p < .001***	WMC negatively associated with MI susceptibility
Zhu et al., 2010	Short-term recall memory	Weschler Memory Scale – Recall (Gong et al., 1989)	Cited Gong et al., 1992: Test-retest reliability *r* = .82	All participants	Pearson’s correlation	r(434) = -.18, p < .001***	Short term recall memory negatively associated with MI susceptibility
Zhu et al., 2010	Short-term recognition memory	Weschler Memory Scale – Recognition (Gong et al., 1989)	Cited Gong et al., 1992: Test-retest reliability *r* = .82	All participants	Pearson’s correlation	r(434) = -.12, p < .05*	Short term recognition memory negatively associated with MI susceptibility
Zhu et al., 2010	Visual perceptual discrimination ability	Motor-Free Visual Perception Test (Colarusso & Hamill, 2003)	Cited Colarusso & Hamill, 2003: Cronbach’s alphas all > .90	All participants	Pearson’s correlation	r(434) = -.29, p < .001***	Perceptual discrimination ability negatively associated with MI susceptibility
Zhu et al., 2010	Visual perceptual discrimination	Change Blindness Test (Rensink et al., 1997)	This study: Cronbach’s alpha = .92	All participants	Pearson’s correlation	r(434) = -.23, p < .001***	Perceptual discrimination ability negatively associated with MI susceptibility
Zhu et al., 2010	Auditory perceptual discrimination	Tone Discrimination Test (Zatorre, 2003)	This study: Cronbach’s alpha = .85	All participants	Pearson’s correlation	r(434) = -.23, p < .001***	Perceptual discrimination ability negatively associated with MI susceptibility
Zhu et al., 2010	Face recognition	Cambridge Face Memory Test (Duchaine & Nakayama, 2006)	This study: Cronbach’s alpha = .85	All participants	Pearson’s correlation	r(434) = -.16, p < .01**	Face recognition memory negatively associated with MI susceptibility
Zhu et al., 2010	Facial expression recognition	Facial Expression Recognition Test (Wang & Markham, 1999)	This study: Cronbach’s alpha = .83	All participants	Pearson’s correlation	r(434) = -.19, p < .01**	Facial expression recognition negatively associated with MI susceptibility
Calvillo, 2014	Working memory capacity	Operation Span (Unsworth et al., 2005)	Cited Unsworth et al., 2005: Test-retest reliability *r* = .83; Cronbach’s alpha = .78	All participants	Pearson’s correlation	r(74) = -.25, p < .05*	WMC negatively associated with MI susceptibility
Calvillo, 2014	Visual perceptual discrimination	Group Embedded Figures Test (Witkin et al., 1971)	Cited Kepner & Neimark, 1984: Test-retest reliability *r* = .78 to .92; Cited Carter & Loo, 1980 Cronbach’s alpha = .86	All participants	Pearson’s correlation	r(74) = -.29, p < .05*	Perceptual discrimination ability negatively associated with MI susceptibility
Nichols & Loftus, 2019	Analytical reasoning	3-item Cognitive Reflection Task (Frederick, 2005)	Not reported	All participants	Pearson’s correlation	r(295) = -.07, p > .05	No significant association between analytical reasoning and MI susceptibility
Farina & Greene, 2020	Autobiographical memory specificity	Shortened Autobiographical Memory Specificity Task (de Decker et al., 2003)	Not reported	All participants (Model with Time of memory test (immediate and 7 days), and visual perceptual load imposed by EW stimuli (high and low)	ANOVA	Time 1: F(2,263) = 13.32, p = .001***, np^2^ = .05 Time 2: F(2,207) = 0.62 p = .43, np^2^ = .003	AMS reduced misinformation susceptibility for leading questions at Time 1 but did not affect MI susceptibility after 7 days at Time 2
Greene et al., 2020	Working memory capacity	Operation Span (Unsworth et al., 2005)	This study: Split-half reliability *r* = .86 Cited Unsworth et al., 2005: Test-retest reliability *r* = .83; Cronbach’s alpha = .78	All participants (Model with visual perceptual load imposed by EW stimuli (high and low)	ANCOVA	Main effect: F(2,198) = 1.59, p = .21, np^2^ = .01 Interaction with perceptual load: F(2,198) = 6.03, p = .01**, np^2^ = .03	WMC did not affect misinformation susceptibility overall but did moderate the effects of high perceptual load on MI susceptibility.
Greene et al., 2020	Verbal intelligence	Wordsum (Thorndike & Gallup, 1944)	This study: Cronbach’s alpha = .72	All participants (Model with visual perceptual load imposed by EW stimuli (high and low)	ANCOVA	Main effect: F(2,198) = 4.80 p = .03*, np^2^ = .02 Interaction with perceptual load: F(2,198) = 0.02, p = .90, np^2^ = .00	Higher intelligence associated with reduced misinformation susceptibility overall, but did not impact effects of perceptual load
Greene et al., 2020	Analytical reasoning	7-item Cognitive Reflection Task (Frederick, 2005; Toplak et al., 2014)	This study: Cronbach’s alpha = .68	All participants (Model with visual perceptual load imposed by EW stimuli (high and low)	ANCOVA	Main effect: F(2,198) = 6.21, p = .01*, np^2^ = .03 Interaction with perceptual load: F(2,198) = 4.25, p = .04*, np^2^ = .02	Higher analytical reasoning associated with reduced misinformation susceptibility. Higher analytical reasoning also moderated the effects of high perceptual load on misinformation susceptibility.
Greene et al., 2021	Verbal intelligence	Wordsum (Thorndike & Gallup, 1944)	This study: Cronbach’s alpha = .72^†^	All participants (Model with misinformation delivery type)	ANCOVA	Main effect: F(1,1157) = 0.15, p = .70, np^2^= .00 Interaction with misinformation delivery type F(1,1157) = 0.35, p = .55, np^2^= .00	No main effect of verbal intelligence on misinformation susceptibility or control item accuracy. No interaction effects with misinformation delivery type.

Study = Citation; Cognitive function = Name of the cognitive function measure; Task = Task used to measure the cognitive function; Task reliability = Internal reliability calculated with the sample in the included study, internal reliability cited by the authors of the included study that was reported elsewhere. Group analysis = Whether all participants analysed or whether they were split into sub-groups; Stat. test = Statistical test used to measure the association between MI susceptibility and individual differences in a cognitive function; Results = Statistical results (* = significant at p < ** = significant at p < .01; *** = significant at p < .001); Finding description = Verbal description of the main findings

†Reliability calculated from raw data for inclusion in the present report

#### Memory abilities

As the misinformation effect is a distortion impacting the accuracy of our recollections, it is unsurprising that the relationship between eyewitness misinformation susceptibility and individual memory abilities is the most widely investigated in this literature. Six of the nine included studies included in this review investigated individual differences in a memory function.

The most frequently investigated mnemonic function was working memory capacity (Calvillo, 2014; Greene et al., 2020; Jaschinski & Wentura, 2002; Parker et al., 2008; Zhu et al., 2010), defined as the number of items that can be held in short-term memory to problem-solve, in the face of interference from irrelevant stimuli (Wilhelm et al., 2013). Four of these studies used an Operation Span task to measure working memory capacity (Calvillo, 2014; Greene et al., 2020; Jaschinski & Wentura, 2002; Parker et al., 2008), which requires participants to memorise a series of letters, while simultaneously solving arithmetic problems (Unsworth et al., 2005). One study used a two-back task (Zhu et al., 2010) that asks participants to remember if a letter shown is the same letter, or a different letter, from that shown two letters previously (Owens et al., 2018). It should be noted that despite both tasks being used to measure working memory capacity (Wilhelm et al., 2013), they are only weakly correlated and should be considered as distinct indicators of working memory ability, with the two-back task being linked to working memory updating ability as well as capacity (Schmiedek et al., 2014). All studies that utilised the Operation Span task found that working memory capacity had small to moderate negative associations with eyewitness misinformation susceptibility (*r* = -.35; *pr* = -.28; *r* = -.25; *np*^*2*^ = .03). Performance in the two-back task was also negatively associated with misinformation susceptibility (*r* = -.17) and source-monitoring errors (*r* = -.13), though these associations are weaker than those in studies that utilised the Operation Span task.

An interesting finding to emerge with regard to working memory capacity is that, while it is correlated with reduced susceptibility to misinformation effects, there is some evidence that working memory capacity does not improve eyewitness accuracy for control items (Jaschinski & Wentura, 2002; Greene et al., 2020). Furthermore, two of the included studies found that working memory capacity only reduced misinformation effects in specific situations. Parker et al. (2008) found that working memory capacity was significantly associated with a reduction in misinformation susceptibility (*r* = -.38), but only in situations where participants were led to believe that they had received a cognitive-enhancing drug and were experiencing a placebo effect. In situations where participants were not experiencing this placebo effect, working memory capacity was not significantly associated with a reduction in misinformation susceptibility at all. Greene et al. (2020) also found evidence for this circumstantial contribution of working memory capacity to misinformation resistance: in this study, there was no significant main effect on misinformation item accuracy overall but working memory capacity did reduce susceptibility to misinformation in conditions where the eyewitness stimuli imposed a high level of visual perceptual load on participants (*np*^*2*^* =* .03). Tentatively, findings from these two studies could indicate that working memory capacity only plays an important role in eyewitness misinformation susceptibility in scenarios where the individual is more likely to require increased focus when completing the eyewitness paradigm, or in situations where cognitive resources are compromised by a circumstantial factor. Under these circumstances, those with higher working memory capacity may be better equipped to retain the necessary information from the eyewitness scene compared to those with lower levels of capacity.

Short-term memory had a small negative correlation with eyewitness misinformation susceptibility in Zhu et al. (2010), in both recall (*r* = -.18) recognition (*r* = -.12) subscales of the Weschler Memory Scale – Chinese adaptation (Gong et al., 1989). However, neither short-term recall nor recognition were associated with source-monitoring errors. Short-term recognition of faces on the Cambridge Face Memory Test (Duchaine & Nakayama, 2006) and recognition of facial expressions on the Facial Expression Recognition Test (Wang & Markham, 1999) also had small negative correlations with misinformation susceptibility (*r* = -.16 and *r* = -.19, respectively), but were significantly correlated with source-monitoring errors (both *r* = -.15) unlike the WMS short-term memory tests. It is unclear why the association only seems to be present for the facial memory tests, and not the WMS tests, which asked participants to remember images of objects.

Only one study looked at the effects of long-term memory ability in relation to eyewitness misinformation susceptibility, and this was levels of specificity in autobiographical memory recollections (Greene et al., 2020). Interestingly, this study found that individuals with more specific autobiographical memories did not have reduced susceptibility to misinformation after a 1-week delay between witnessing the stimuli and recollection, but that it did decrease susceptibility to leading questions shortly after exposure to the eyewitness stimuli. With this finding, it should be noted that this was the only study included in this review that looked at the relationship between individual differences in cognitive function and misinformation susceptibility across different time points. All other included studies only looked at the contribution of cognitive abilities after a short delay from exposure to the eyewitness stimuli.

#### General intelligence and reasoning

Four studies investigated the relationship between eyewitness misinformation susceptibility and individual differences in intelligence or reasoning ability. Across these studies, there were five tasks measuring performance in standardised IQ tests, and two tasks measuring analytical reasoning ability. The exact constructs tapped into by intelligence and reasoning tests are debated, but standardised IQ tests typically aim to produce a score that is an index of individual differences across many cognitive processes (e.g., executive functioning, higher-order reasoning, frontal lobe functioning), rather than a specific cognitive function in itself (Kovacs & Conway, 2019). Typically, the types of functions being measured by intelligence tests can be split into non-verbal measures that do not depend upon prior knowledge to solve, and verbal tasks that do rely on acquired skills to solve (Schneider & McGrew, 2012). Significant associations between intelligence and ability in areas of higher order cognition and misinformation susceptibility may reflect the role of general cognitive ability in reducing the likelihood of episodic memory distortions.

Two non-verbal measures of intelligence were linked with a significant reduction in eyewitness misinformation susceptibility: Raven’s Advanced Progressive Matrices (RAPM) measuring abstract reasoning (Raven et al., 1998) and a non-verbal subscale of a revised Chinese version of the Wechsler Adult Intelligence Scale (WAIS-RC). The WAIS-RC contained three tasks indexing visual perception, visual-motor co-ordination, and abstract reasoning (Gong et al., 1992). Both tasks were administered in the same study (Zhu et al., 2010), and found small to moderate negative correlations with overall eyewitness misinformation susceptibility (*r* = -.35 for the RAPM; *r* = -.29 for the WAIS-RC performance), meaning the factors measured by these non-verbal IQ tests could potentially provide some level of protection against misinformation distortions to episodic memory. In addition to measuring the number of misinformation items reported overall, Zhu et al. (2010) also measured how many individuals remembered having seen the misinformation detail in the original eyewitness stimuli, to assess if the participant had made a source-monitoring error (i.e., identifying the source of the misinformation as the eyewitness stimulus, rather than information they encountered after the fact). These non-verbal IQ measures had smaller, but still significant, correlations with source-monitoring errors (*r* = -.23 for the RAPM, *r* = -.18 for the WAIS-RC performance). In addition to these non-verbal standardised IQ measures, two spatial reasoning tasks were negatively associated with habitual susceptibility to misinformation in another study (Tomes & Katz, 1997). These tasks were the Card Rotation and Paper Folding tasks (Ekstrom et al., 1976), which indexed mental imagery rotation and visuo-spatial manipulation ability, respectively. Better performance in both tasks was associated with a small decrease in susceptibility to misinformation effects across three separate eyewitness events (*r* = -.20).

There were two standardised verbal IQ measures employed in the included studies; the verbal subscale of the WAIS-RC, which contained three tasks measuring general knowledge, verbal reasoning, and attention (Gong et al., 1992), was measured in one study (Zhu et al., 2010), and the Wordsum vocabulary task (Thorndike & Gallup, 1944), which is strongly associated with overall IQ scores, was measured in two studies (Greene et al., 2020; Greene et al., 2021). Performance in the WAIS-RC verbal was significantly associated with reduced misinformation effects (Zhu et al., 2010), but the correlation was much weaker than that found for the non-verbal measures in the same study (*r* = -.13). Furthermore, despite non-verbal intelligence tasks being correlated with source-monitoring errors, this was not the case for the WAIS-RC verbal subscale. The correlation between the WAIS-RC verbal and source-monitoring errors was not significant. Those who performed better in the Wordsum task (Greene et al., 2020) did show a decreased level of susceptibility to the misinformation effect (*η*_*p*_^*2*^ = .02), but this effect may be explained by an improvement in global memory performance, as accuracy for control items was also impacted by Wordsum scores with a larger effect size (*η*_*p*_^*2*^ = .07). However, this finding was not replicated in Greene et al. (2021), which found that there was no significant main effect of Wordsum scores on misinformation endorsement or control item accuracy, and there was no interaction between Wordsum scores and any of the six types of misinformation delivery method used (doctored photographs, elaborate leading questions, simple leading questions, chronological narrative, scrambled narrative, and fill-in-the-blanks narrative). Thus, findings based on verbal intelligence measures tended to be less consistent and found weaker effects than those based on non-verbal intelligence measures.

Two studies measured verbal analytical reasoning ability using a Cognitive Reflection Task (CRT; Frederick, 2005), assessing whether participants engage in effortful deliberation when problem-solving, or whether they provide their initial “gut” reaction without engaging in reasoning. Interestingly, performance in the CRT has been associated with a better ability to discern between true and fake news (Pennycook & Rand, 2019), and also is associated with a decreased likelihood of having seen any novel news story before, be it true or false (Greene & Murphy, 2020). This indicates that better analytical reasoning, as measured by the CRT, may increase scrutiny over novel information and help us to better distinguish between true and false information. However, it is unclear whether this advantage of better analytical reasoning extends to misinformation resistance in an eyewitness context. Like the Wordsum task in Greene et al. (2020), better CRT performance indicated increased memory accuracy for both misinformation (*η*_*p*_^*2*^ = .03) and control items (*η*_*p*_^*2*^ = .02). However, the CRT was not associated with accuracy for misinformation items, control items, or source-monitoring errors in a different study (Nichols & Loftus, 2019). However, it should be noted that in this study, the original, and shorter, three-item version of the CRT was used, and results were split into number of intuitive responses versus deliberate responses, rather than just summing the correct deliberate CRT responses as in Greene et al. (2020). Thus, methodological differences may account for the differing findings between the two studies that included a measure of analytical reasoning ability.

#### Perceptual abilities

Two studies included in this review investigated associations between individual differences in perceptual abilities and eyewitness misinformation susceptibility (Calvillo, 2014; Zhu et al., 2010). More specifically, these studies used tasks measuring perceptual discrimination, an ability in sensory processes and categorisation that allows us to detect similarities or differences between external stimuli (Laurent et al., 2006). Perceptual discrimination is an important function that is linked with performance in several other cognitive domains such as reasoning and problem solving (Salvucci & Anderson, 2001), and working memory (Covey et al., 2019; Berry et al., 2009).

Four measures were used to assess perceptual discrimination ability: three in the visual domain: the Motor-Free Visual Perception Test (MFVP; Colarusso & Hammil, 2003), Change Blindness Test (Rensink et al., 1997), and the Group Embedded Figures Test (GEFT; Witkin et al., 1971), and one in the auditory domain: the Tone Discrimination Task (Zatorre, 2003). Better performance in each of these tasks being was associated with small to moderate reductions in the misinformation effect. Interestingly, while the MFVP test (*r* = -.14), and the Tone Discrimination task (*r* = -.20) were also associated with decreased source-monitoring errors, the Change Blindness task was not (Zhu et al., 2010). No information regarding source-monitoring errors and GEFT performance was available (Calvillo, 2014). No information on the relationship between perceptual discrimination ability and eyewitness memory accuracy for control items is available in either study, so it is unclear if perceptual abilities improve global eyewitness memory performance, or if this association is specific for reducing the misinformation effect.

## Discussion

This review set out to identify which cognitive abilities have been associated with susceptibility to the misinformation effect in eyewitness scenarios in adults, and the nature of any associations. Twenty-three measures of individual differences in cognitive ability were identified in this regard. These findings were classified into three broad categories based on the type of cognitive function being measured: general intelligence and reasoning ability, perceptual abilities, and memory abilities. The vast majority of findings indicated that those who possessed higher levels of cognitive ability, across all three categories, were significantly less susceptible to misinformation effects in eyewitness scenarios. The exception to this was verbal intelligence and verbal reasoning ability, which found small significant associations in some cases (Greene et al., 2020; Zhu et al., 2010), but did not reach significance in others (Greene et al., 2021; Nichols & Loftus, 2019). Overall, it seems that a number of cognitive abilities, such as working memory capacity, non-verbal intelligence, and perceptual discrimination abilities, have been linked with a reduced misinformation effect in multiple studies. However, this review also highlighted significant limitations within this body of research, largely due to the small number of relevant studies, as well as the heterogeneity of how the misinformation effect was defined across included studies. Thus, while the main conclusions from this review will be discussed, we also caution that any assertions are tentative, and a lot of questions about the link between cognitive abilities and eyewitness misinformation susceptibility remain unanswered.

## Overview of findings

Measures of general intelligence and reasoning ability assessed broad cognitive functioning skills typically employed for higher-level processing and problem-solving (Kovacs & Conway, 2019). There were some discrepancies in findings on the relationship between intelligence and eyewitness misinformation susceptibility, which appeared to be based on whether the tasks employed were non-verbal or verbal in nature. Such a discrepancy makes sense, given the long-standing distinction between fluid intelligence, which is based on non-verbal abstract reasoning, and crystallised intelligence, which relies on existing, often verbally based knowledge (Cattell, 1987). Non-verbal measures (Tomes & Katz, 1997; Zhu et al., 2010) had a stronger link with reduced misinformation effects overall compared to verbal measures (Greene et al., 2020; Greene et al., 2021; Nichols & Loftus, 2019; Zhu et al., 2010). This might be expected, given that fluid intelligence has been found to impact long-term memory accuracy and retention to a much larger extent than crystallised intelligence (Alexander & Smales, 1997; Unsworth, 2010), and that the improvement to memory associated with crystallised intelligence could even be accounted for by the investment of fluid intelligence during verbal problem-solving tasks (Martinez, 2019).

From the findings in this review, it appears that intelligence, particularly non-verbal, fluid intelligence, is related to eyewitness misinformation susceptibility. However, the question still remains as to why this might be the case. Zhu et al. (2010), who reported two measures of non-verbal intelligence, and one measure of verbal intelligence, found that better performance in the non-verbal measures resulted not only in moderate reductions in misinformation errors, but also reduced source-monitoring errors. However, no relationship between the verbal IQ measure and source-monitoring errors was found. Nichols and Loftus (2019) also found no relationship between verbal analytical reasoning ability and misinformation effects nor source-monitoring errors.

Tentatively, we might say that it appears that fluid intelligence and abstract reasoning may contribute to source-monitoring processes during an eyewitness memory test, thus reducing misinformation effects, whereas crystallised intelligence does not. In another study, a verbal intelligence measure and a verbal analytical reasoning measure were both associated with increased memory accuracy for control items, as well as for misinformation items, indicating that crystallised intelligence is associated with an improvement to global episodic memory, as opposed to having a specific role in resisting misinformation (Greene et al., 2020). However, this finding was not replicated by Greene et al. (2021), where there was no main effect of verbal intelligence on eyewitness memory accuracy for misinformation or control items. None of the studies with non-verbal intelligence measures provided any associations with global episodic memory, so it is unclear if this overall eyewitness accuracy increase would be linked to fluid intelligence as well. More research investigating not only if certain cognitive abilities are linked to eyewitness misinformation susceptibility, but also how they reduce susceptibility, would enhance our understanding in situations like this. For example, future studies in this area should consider investigating at which stage of an eyewitness misinformation paradigm, the cognitive ability provides protection from distortion. For example, is the ability linked with improved encoding of the original memory? Does the ability increase an individual’s likelihood to detect a discrepancy between the original memory and the misinformation upon exposure? Or does the cognitive ability contribute to source-monitoring ability, improving the chances that the correct memory detail will be remembered over the misinformation?

Perceptual processes have long been considered as an important factor in preventing memory distortions, as the strength of initial early encoding of a memory is thought to be a contributing factor to whether misleading suggestions are incorporated during recollection (Okado & Stark, 2005), and perceptual disruptions during encoding are thought to also increase misinformation effects (Laney & Loftus, 2010). Thus, it would make sense for individual perceptual abilities to be related to eyewitness misinformation susceptibility, and the evidence from this review supports this claim. All four perceptual ability measures were negatively associated with eyewitness misinformation susceptibility. However, it should be noted that three of these four measures came from Zhu et al. (2010), with one measure of visual field independence from Calvillo (2014), so these findings require further replication and support. Furthermore, it is unclear whether perceptual abilities improve source-monitoring ability based on the findings from these two studies. In Zhu et al. (2010), performance in the Motor-Free Visual Perception battery and Tone Discrimination task were both negatively associated with source-monitoring errors, but the change blindness task was not. Calvillo (2014) did not include a measure of source-monitoring errors. It is also unclear whether perceptual abilities improve global eyewitness memory performance, as no associations between control item accuracy and these measures were made available in either study. This further highlights the need for studies to consider individual differences at each stage of the eyewitness paradigm, to provide a more detailed understanding of the cognitive factors underpinning misinformation distortions.

Working memory capacity was the most widely investigated cognitive ability in this review (Calvillo, 2014; Greene et al., 2020; Jaschinski & Wentura, 2002; Parker et al., 2008; Zhu et al., 2010), with all results finding that those with higher working memory capacity were better able to resist the effects of post-event misinformation to memory. Interestingly, data from both Jaschinski and Wentura (2002) and Greene et al. (2020) suggest that working memory capacity makes a unique contribution to protecting against misinformation, rather than simply improving global episodic memory. This indicates that the strength of encoding of the original memory may not be the only important contributing factor to misinformation susceptibility in eyewitness scenarios. It has been suggested that the influence of individual difference factors on misinformation susceptibility may be negligible as those who deeply encode the original eyewitness event will also deeply encode the post-event misinformation, thus cancelling out the benefits of these abilities (Patihis, 2018). However, these two findings on working memory capacity tentatively indicate that this cognitive ability contributes to more than just improved encoding ability and may help participants to identify and discard post-event information upon exposure, through discrepancy detection (Tousignant et al., 1986). However, further research is needed to clarify whether working memory capacity may influence discrepancy detection rates, as despite the fact that it can play an important role in reducing misinformation effects, explicitly measuring whether discrepancy detection occurred is not frequently implemented in these paradigms (Butler & Loftus, 2018).

It is likely that working memory capacity may also influence source-monitoring during an eyewitness memory test. It has been noted that performance in span tasks measuring working memory capacity, which is the measure that four out of five studies in the review, is dependent on the ability to monitor the source of information and determine whether it is task-relevant or not (Shipstead et al., 2014). Furthermore, working memory capacity has also been linked with performance in wordlist false memory paradigms, and it has been found that source-monitoring ability fully mediates this association using this paradigm (Ball et al., 2022; Unsworth & Brewer, 2010). However, as there are different underlying mechanisms to the memory distortions induced by wordlist and eyewitness episodic memory misinformation paradigms (Ost et al., 2013), it is worth explicitly investigating whether source-monitoring explains the working memory and misinformation effect link identified in this review.

Only one study (Zhu et al., 2010) looked at the effects of short-term memory on eyewitness misinformation susceptibility, with two measures of short-term recall and recognition for objects, and two short-term memory tests for faces. Higher scores on each of these measures was associated with reduced misinformation susceptibility. Unusually, source-monitoring errors were not associated with the short-term recall and recognition tests for objects but were associated with facial recall and recognition abilities. Based on the limited amount of data, it is unclear why short-term memory for faces is specifically related to source-monitoring, especially as the questions asked in this study pertained to events of the eyewitness stimuli rather than facial recognition. Still, the effects of all the short-term memory measures were quite small compared to other abilities investigated in this study with regard to reducing misinformation susceptibility. Again, as the authors note, this indicates cognitive factors other than just general memory ability influence misinformation susceptibility.

Similarly, only one study looked at the effects of a long-term memory measure, which was the specificity and detail of autobiographical memories (Farina & Greene, 2020). Interestingly, despite autobiographical memory specificity not being associated with misinformation susceptibility after a delay of 1 week, it was found to reduce susceptibility to leading questions shortly after viewing the eyewitness stimuli. This study was the only one in this review to look at the effects of these cognitive ability factors after a delay of longer than a few minutes, so it prompts the recommendation that other cognitive abilities need to be looked at across different time intervals in case their impact wanes over time. This finding regarding autobiographical memory specificity is also interesting when considering previous findings that those with highly superior autobiographical memory were more prone to misinformation effects in an eyewitness paradigm (Patihis, 2018). However, as this is only one study, more research is needed into the influence of individual differences in autobiographical memory, and whether they provide protection from misinformation in any capacity.

## The role of cognitive abilities under different circumstances

When considering the findings of this review, it is important to highlight that only nine studies investigating the relationship between eyewitness misinformation susceptibility and individual differences in cognitive ability were identified. This limited number of studies is not overly surprising, as it has been noted that experimental manipulations of circumstantial factors that may increase or decrease misinformation susceptibility is a more popular approach (Nichols & Loftus, 2019), mainly due to the fact it has more direct practical applications for eyewitness contexts. Interestingly, some findings from this review indicate that looking at individual difference factors in cognitive functioning may moderate the influence of different circumstantial factors on misinformation susceptibility. Three of the studies in this review identified potential interactions between circumstantial factors and individual difference factors, each finding that cognitive abilities only appeared to reduce eyewitness misinformation susceptibility under specific conditions. As mentioned previously, autobiographical memory specificity only had a significant effect on misinformation effect rates when asked leading questions shortly after viewing the eyewitness stimuli, but not when asked to recall details of the event 1 week later (Farina & Greene, 2020).

The other two findings related to the role of working memory capacity. In Greene et al. (2020) working memory capacity reduced misinformation susceptibility only for participants who viewed an eyewitness video that imposed a high level of visual perceptual load. This indicates that when attentional resources are compromised during encoding, those with higher working memory capacity may be less impacted than those with lower levels of capacity. Similarly, Parker et al. (2008) found that the effects of working memory capacity on reducing misinformation susceptibility were only significant in circumstances where participants believed they had received a cognitive-enhancing drug, inducing a placebo effect. This placebo effect may have resulted in the participants exerting more cognitive effort during the eyewitness misinformation paradigm, with those with higher levels of working memory capacity being more equipped for this specific situational demand. Studies that have examined the role of working memory capacity in the context of other types of false memory have found similar situational effects, like that of Gerrie and Garry (2007), which identified that working memory capacity only reduced false memories for details that are important to the central event in a scene, but not for more minor details. Thus, findings from this review provide a good case for not only looking at differences in eyewitness misinformation effects in different circumstances, but also looking at how individual differences in cognitive ability may moderate these effects, in order to better understand which cognitive functions may contribute to resisting misinformation distortions to episodic memory.

Another area for further study would be investigating interactions between age and different cognitive abilities on eyewitness susceptibility to misinformation. Inclusion criteria for this review specified that studies were required to have been conducted solely with adult populations. The exclusion of studies with children stemmed from the challenge of comparing findings between adult and child cohorts due to developmental disparities in cognitive function. However, we can tentatively consider the findings of this review next to similar systematic reviews on individual differences in misinformation susceptibility in children (Bruck & Melnyk, 2004; Klemfuss & Olaguez, 2020). Although these reviews did identify some studies presenting significant negative associations between levels of misinformation susceptibility and scores in cognitive ability, findings were mixed and inconsistent in both reviews, with several studies finding no link between cognitive ability and susceptibility to misinformation in children. Thus, our review demonstrated more consistent associations in studies conducted with adults. Discrepancies between these findings could be attributed to the fact that Bruck and Melnyk (2004) and Klemfuss and Olaguez (2020) included studies with more varied misinformation methodologies (e.g., trait suggestibility, rich false memory), that the types of cognitive tasks administered to children differed from those in our review with adults, or potential file drawer issues on studies with adults wherein non-significant findings were not published. Alternatively, it could indicate a genuine distinction wherein cognitive abilities hold less sway over misinformation susceptibility during childhood compared to adulthood.

We cannot draw any strong inferences regarding age effects on the link between cognitive ability and misinformation susceptibility from the studies included in this review. Even though the inclusion criteria specified that adults of all ages could be included, the samples in all studies where age demographic information was provided were concentrated on younger adults (aged under 35 years). Older adults have been found to be more prone to the misinformation effect than younger adults (Umanath et al., 2019; Wylie et al., 2014), which has been theorised to be due at least in part to a decline of certain cognitive abilities with increased age, particularly abilities centralised in frontal lobe functioning (Davis & Loftus, 2005; Meade et al., 2012; Roediger & Geraci, 2007). In future research, it would be worth investigating the impact of specific cognitive abilities on misinformation susceptibility throughout adulthood and across the lifespan to identify the stages at which certain cognitive abilities generally provide the most protection and how they provide this protection (e.g., better source-monitoring ability in younger adults).

Finally, another circumstantial factor to note in this body of research is the mode of data collection, given the ever-increasing shift towards running psychological studies online (Buhrmester et al., 2018), including studies assessing eyewitness identification and memory accuracy (Kovera & Evelo, 2021). This shift to online studies is reflected in the included articles in this systematic review, with the three most recent studies running their studies entirely online, while all prior studies collected data in a laboratory setting on a university campus. Moving studies online brings several benefits, including increased accessibility to more diverse and representative samples and the ability to gather larger samples in a short space of time using paid crowd-sourcing sites such as Prolific or Amazon’s Mechanical Turk, thus increasing the statistical power of analyses (Newman et al., 2021; Peer et al., 2022). While there is evidence that suggests that these samples are of comparable quality and similar to those collected in the lab using a large range of social science methodologies (Casler et al., 2013; Clifford et al., 2015), there are also some conflicting findings suggesting that quality of data obtained from online participants, particularly those recruited through paid crowdsourcing sites, has decreased in recent years (Chmielewski & Kucker, 2020). Thus, it is worth considering how the experimental context may impact findings using specific paradigms, like those implemented in eyewitness misinformation studies, to ensure that they are of an equal and comparable standard.

To our knowledge, no research has compared the quality of data in online versus in-person eyewitness misinformation paradigms. We argue that this is a critical gap in the literature, especially for studies looking at the link between misinformation effects and cognitive factors, as these studies assess variables that are heavily intertwined with attentional processes (Lane, 2006; Zaragoza & Lane, 1998). A lack of control over environmental factors when administering these studies remotely could plausibly confound results. It is possible that some discrepancies in findings in this review could be attributed to the mode of data collection, (e.g., Greene et al., 2020, found a significant association between misinformation susceptibility and analytical reasoning in a CRT when conducted online, while Nichols & Loftus, 2019, did not find this association with a study conducted in a laboratory setting). However, it is impossible to make anything but speculative comments about this without further research comparing online versus offline eyewitness memory research. Thus, a recommendation for future studies is to investigate whether the impact of these cognitive abilities on eyewitness misinformation susceptibility differs depending on the context in which the data is collected

## Limitations

The findings from this review are limited in several ways. The wide range of variability in how the misinformation effect was studied and measured made the generalisability across the different variables only possible through narrative syntheses, preventing any statistical summaries. Also, it should be noted that the scope may have been limited by only including peer-reviewed publications, especially given the small effect sizes of individual difference associations. Any findings that may have failed to reach the *p* < .05 significance may not have been published, with this potential file drawer issue not being ruled out. This concern is only exacerbated by the risk of bias appraisals conducted on the included findings in this review, which found that only one of the nine studies had published a pre-registered analysis plan (Greene et al., 2021).

Another notable limitation of the findings from this review pertains to the internal reliability of the measurements used, particularly in measurements of individual levels of susceptibility to the misinformation effect. While good to excellent reliability estimates were reported for the majority of the cognitive tasks used in the included studies, no study reported information regarding reliability estimates for the eyewitness memory tests employed to measure susceptibility to misinformation. This is likely because utilizing the eyewitness memory test either employed within-subject designs with counterbalanced items or featured a limited number of items rendering the calculation of reliability scores to be unfeasible (Nichols & Loftus, 2019). Consequently, as there is a lack of reliability estimates for the misinformation effect measurements, it is important that we consider these findings with caution. While all but one of the correlations between cognitive ability and misinformation susceptibility were statistically significant, we cannot rule out that the magnitude of the relationships could have been attenuated by issues of reliability for misinformation effect measurements (Spearman, 1904).

Also, the specificity of the research question for this article meant that non-performance-based outcome measures of cognitive abilities or functions were excluded from the review, such as Need for Cognition (Bailey et al., 2021) or Vividness of Visual Imagery (Tomes & Katz, 1997), which have been studied in relation to eyewitness memory and misinformation before. However, generally, the effects that these experiential traits have is quite minimal (Patihis, 2018), and may be more reflective of personal tendencies rather than an actual mechanism that provides protection against resisting misinformation. It is also important to emphasise that the associations between the included outcome measures and misinformation susceptibility in eyewitness scenarios probably do not extend to other types of false memories induced through misinformation, as very little overlap has been found between different types of false memory paradigms (Nichols & Loftus, 2019), likely indicating that there are different underlying mechanisms at play.

## Conclusions

Investigating links between individual differences in cognitive functions and eyewitness susceptibility to misinformation can help in progressing theoretical frameworks used to explain this phenomenon, and aid in answering the question – are some people more vulnerable to the misinformation effect than others? The main conclusion to be drawn from this review is that cognitive abilities do appear to impact eyewitness misinformation susceptibility in adults, with better performance across different cognitive tasks being associated with reduced misinformation effects. However, how exactly each of these cognitive functions prevents misinformation from distorting memory remains unclear and requires further investigation. Similarly, some findings from this review highlight how investigating interactions between these cognitive abilities and circumstantial factors may be beneficial in identifying the cognitive mechanisms underpinning misinformation distortions to eyewitness memory. This review concludes that it is likely that while all people are likely prone to the misinformation effect on some level, the current evidence indicates that individual differences in cognitive ability does seem to create at least some variance in how susceptible an individual is to this type of memory distortion.

## Supplementary Information

Below is the link to the electronic supplementary material.

Supplementary file1 (DOCX 556 KB)

## Data Availability

The datasets generated during and/or analysed during the current study are available via the Open Science Framework repository at: https://osf.io/kwh3a/

## Declarations

### Open Practices Statement

The datasets generated and/or analysed during the current study are available via the Open Science Framework repository at: https://osf.io/kwh3a/, and the protocol for this review was pre-registered on PROSPERO before conducting electronic database searches (https://www.crd.york.ac.uk/prospero/display_record.php?RecordID=250947).
