# Supplementary material for: Do cognitive abilities reduce eyewitness susceptibility to the misinformation effect? A systematic review
Source: Psychon Bull Rev. 2024 May 2;31(6):2410–36. doi: 10.3758/s13423-024-02512-5 (PMC11680610; doi:10.3758/s13423-024-02512-5)
Supplement: Supplementary file 1 — Supplementary file1 (DOCX 556 KB) [file 13423_2024_2512_MOESM1_ESM.docx]

**Supplemental materials**

**Electronic database search strings**

***Academic Search Complete (EBSCOhost): 877 search results: Run on 04.05.21. Re-run on 16.01.23***

(DE "EYEWITNESS identification" OR DE "EYEWITNESS accounts" OR DE "EARWITNESS identification" OR DE "WITNESSES" OR Eyewitness* OR Eye-witness* OR Earwitness* OR Ear-witness* OR Witness* OR Bystander OR Spectator*) AND (DE "MISINFORMATION" OR DE "DISINFORMATION" OR DE "LEADING questions (Law)" OR Misinform* OR Mislead* OR Misled OR Misrepresent* OR Misreport* OR Misstate* OR Disinform* OR Inaccur* OR Incorrect OR Fabricat* OR “Leading question*” OR Distort* OR “Source Confusion” OR “Source Attribution” OR “Source Error*”) AND (DE "COGNITIVE ability" OR DE "COGNITIVE Abilities Test" OR DE "AUTOBIOGRAPHICAL memory" OR DE "EPISODIC memory" OR DE "ATTENTION" OR DE "EXECUTIVE function (Neuropsychology)" OR DE "SHORT-term memory" OR DE "SPATIAL memory" OR DE "VERBAL memory" OR DE "CRYSTALLIZED intelligence" OR DE "FLUID intelligence" OR DE "INDIVIDUAL differences" OR DE "PERCEPTION testing" OR Cognit* OR “Individual difference*” OR Abilit* OR Skill* OR Task* OR Memor* OR Attention* OR “Executive Function*” OR Intelligen* OR Percept*)

***PsycINFO (Proquest): 1218 search results: Run on 04.05.21. Re-run on 16.01.23***

((MAINSUBJECT.EXACT("Witnesses") OR Eyewitness* OR Eye-witness* OR Earwitness* OR Ear-witness* OR Witness* OR Bystander OR Spectator*)) AND ((MAINSUBJECT.EXACT("Source Monitoring") OR Misinform* OR Mislead* OR Misled OR Misrepresent* OR Misreport* OR Misstate* OR Disinform* OR Inaccur* OR Incorrect OR Fabricat* OR "Leading question*" OR Distort* OR "Source Confusion" OR "Source Attribution" OR "Source Error*")) AND ((MAINSUBJECT.EXACT("Fluid Intelligence") OR MAINSUBJECT.EXACT("Attention Span") OR MAINSUBJECT.EXACT("Executive Function") OR MAINSUBJECT.EXACT("Visual Attention") OR MAINSUBJECT.EXACT("Perceptual Measures") OR MAINSUBJECT.EXACT("Attention") OR MAINSUBJECT.EXACT("Intelligence Measures") OR MAINSUBJECT.EXACT("Intelligence") OR MAINSUBJECT.EXACT("Individual Differences") OR MAINSUBJECT.EXACT("Cognitive Processes") OR MAINSUBJECT.EXACT("Cognitive Ability") OR MAINSUBJECT.EXACT("Memory") OR MAINSUBJECT.EXACT("Cognitive Assessment") OR MAINSUBJECT.EXACT("Executive Functioning Measures") OR MAINSUBJECT.EXACT("Perception") OR Cognit* OR "Individual difference*" OR Abilit* OR Skill* OR Task* OR Memor* OR Attention* OR "Executive Function*" OR Intelligen* OR Percept*))

***Web of Science [v.5.35]: 1517 search results: Run on 06.05.21. Re-run on 16.01.23***

(TI=(Eyewitness* OR Eye-witness* OR Earwitness* OR Ear-witness* OR Witness* OR Bystander OR Spectator*) OR AB=(Eyewitness* OR Eye-witness* OR Earwitness* OR Ear-witness* OR Witness* OR Bystander OR Spectator*) OR TS=(Eyewitness* OR Eye-witness* OR Earwitness* OR Ear-witness* OR Witness* OR Bystander OR Spectator*) OR AK=(Eyewitness* OR Eye-witness* OR Earwitness* OR Ear-witness* OR Witness* OR Bystander OR Spectator*)) AND (TI=(Misinform* OR Mislead* OR Misled OR Misrepresent* OR Misreport* OR Misstate* OR Disinform* OR Inaccur* OR Incorrect OR Fabricat* OR “Leading question*” OR Distort* OR “Source Confusion” OR “Source Attribution” OR “Source Error*”) OR AB=(Misinform* OR Mislead* OR Misled OR Misrepresent& OR Misreport* OR Misstate* OR Disinform* OR Inaccur* OR Incorrect OR Fabricat* OR “Leading question*” OR Distort* OR “Source Confusion” OR “Source Attribution” OR “Source Error*”) OR TS=( Misinform* OR Mislead* OR Misled OR Misrepresent* OR Misreport* OR Misstate* OR Disinform* OR Inaccur* OR Incorrect OR Fabricat* OR “Leading question*” OR Distort* OR “Source Confusion” OR “Source Attribution” OR “Source Error*”) OR AK=(Misinform* OR Mislead* OR Misled OR Misrepresent* OR Misreport* OR Misstate* OR Disinform* OR Inaccur* OR Incorrect OR Fabricat* OR “Leading question*” OR Distort* OR “Source Confusion” OR “Source Attribution” OR “Source Error*”)) AND (ALL=(Cognit* OR “Individual difference*” OR Abilit* OR Skill* OR Task* OR Memor* OR Attention* OR “Executive Function*” OR Intelligen* OR Percept*))

***Scopus (Elsevier): 1994 search results: Run on 04.05.21. Re-run on 16.01.23***

(TITLE-ABS-KEY(Eyewitness* OR Eye-witness* OR Earwitness* OR Ear-witness* OR Witness* OR Bystander OR Spectator*)) AND (TITLE-ABS-KEY(Misinform* OR Mislead* OR Misled OR Misrepresent* OR Misreport* OR Misstate* OR Disinform* OR Inaccur* OR Incorrect OR Fabricat* OR "Leading question*" OR Distort* OR "Source Confusion" OR "Source Attribution" OR "Source Error*")) AND (ALL(Cognit* OR "Individual difference*" OR Abilit* OR Skill* OR Task* OR Memor* OR Attention* OR "Executive Function*" OR Intelligen* OR Percept*))

**Inter-rater agreement for article screening**

**Table 1: Title and abstract screening agreement**

| CO Yes, MB Yes | CO Yes, MB No | CO No, MB Yes | CO No, MB No | Proportionate Agreement | \| Cohen's Kappa \| \| --- \| \|  \| |
| --- | --- | --- | --- | --- | --- | --- | --- |
| 26 | 48 | 64 | 3387 | 0.9682 | 0.3010 |

**Table 2: Full-text screening agreement**

| CO Yes, MB Yes | CO Yes, MB No | CO No, MB Yes | CO No, MB No | Proportionate Agreement | \| Cohen's Kappa \| \| --- \| \|  \| |
| --- | --- | --- | --- | --- | --- | --- | --- |
| 9 | 7 | 1 | 22 | 0.7949 | 0.5504 |

**Cochrane Risk of Bias 2 Appraisals**

Quality appraisals were conducted using the Cochrane Risk of Bias 2 tool (Sterne et al., 2019). Appraisals were conducted on each relevant result individually but are summarised by paper here for the sake of brevity. All results from the same study were the equivalent in this review. These figures were created using the robvis web application (McGuinness & Higgins, 2020)

**Figure 1: Traffic Light Plot of RoB judgements**

**
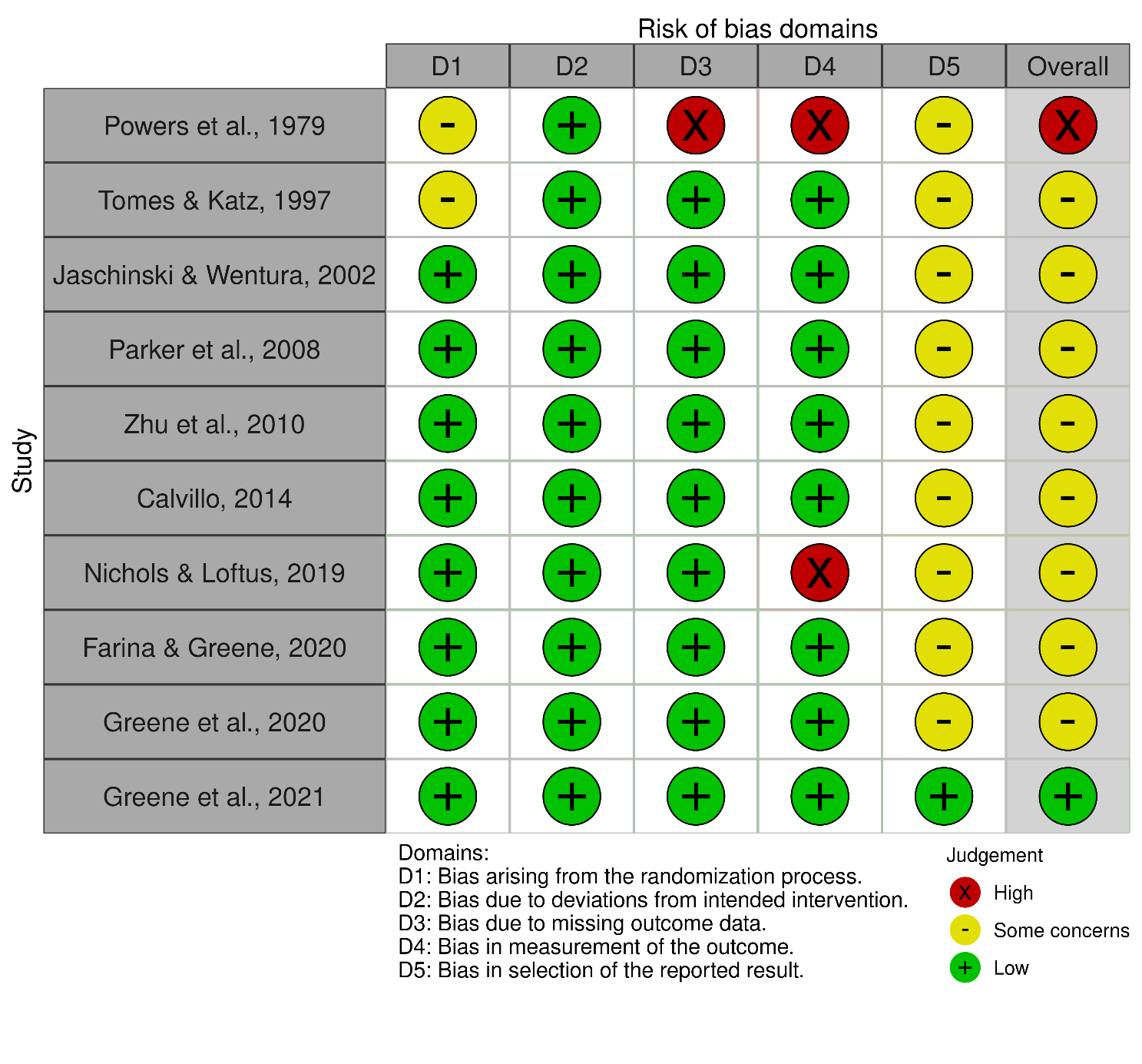
**

**Figure 2:Summary Plot of RoB judgements**

**
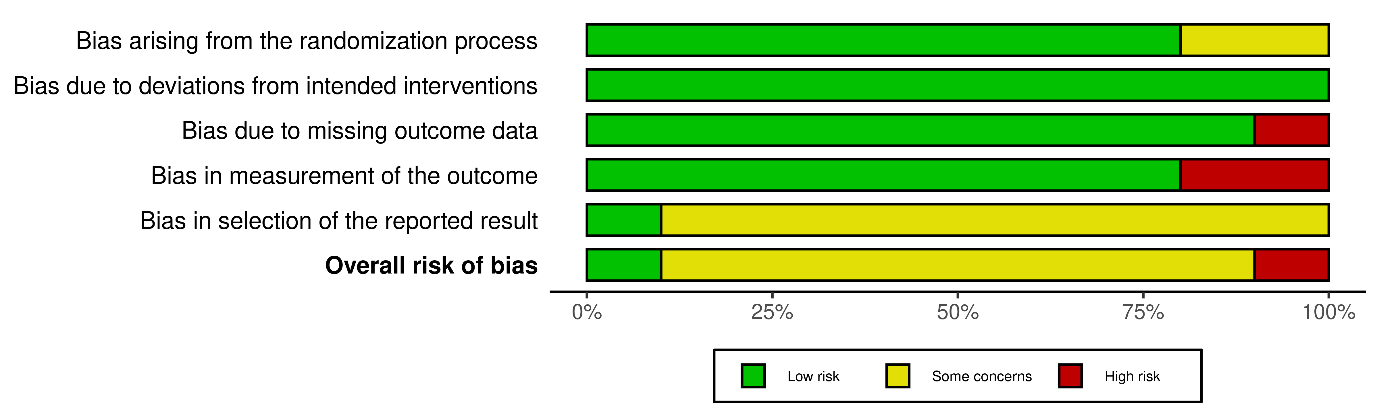
**

**
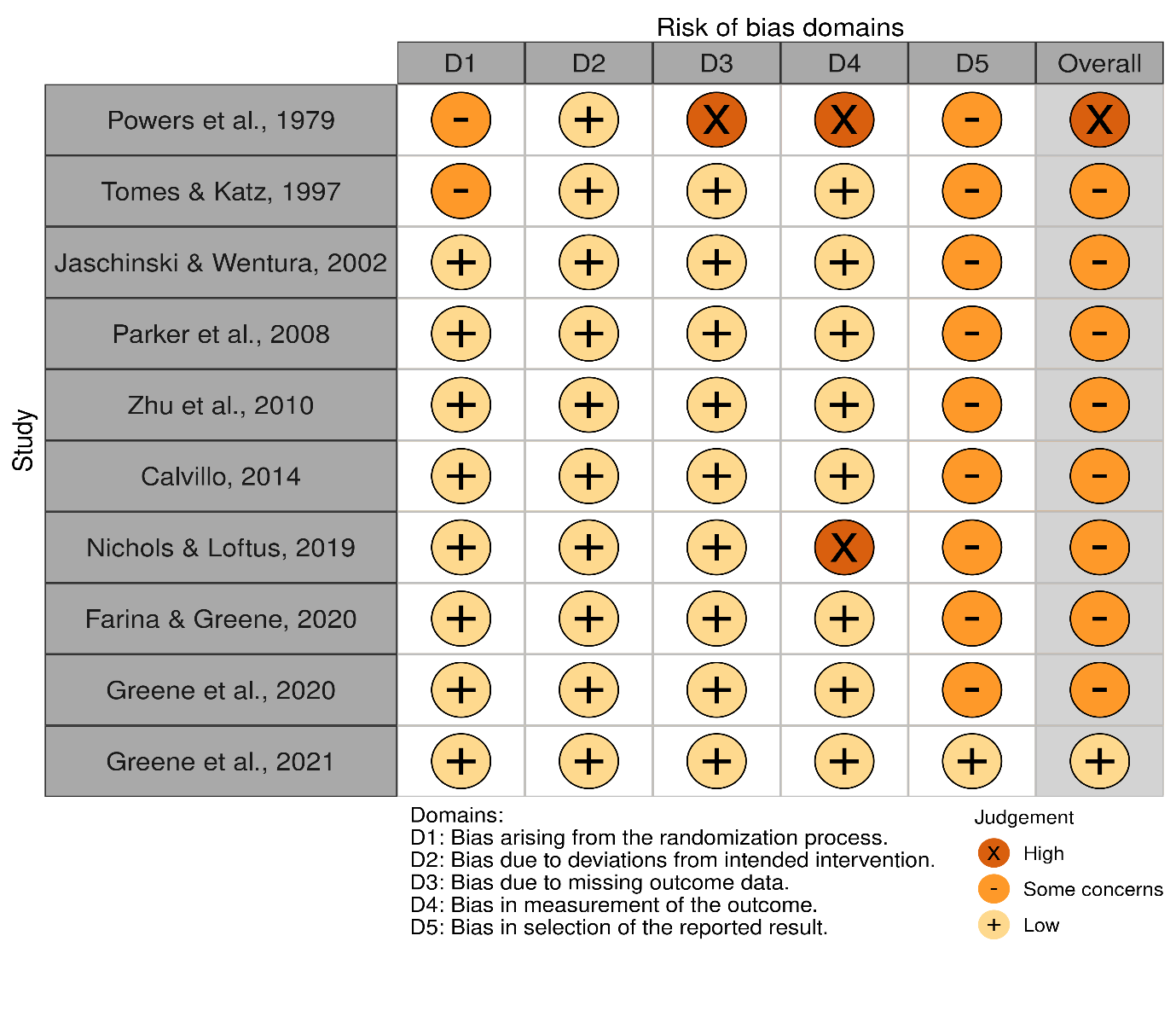
Figure 3: Traffic Light Plot of RoB judgements (Colour-blind friendly)**

**Figure 4:Summary Plot of RoB judgements (Colour-blind friendly)**

**
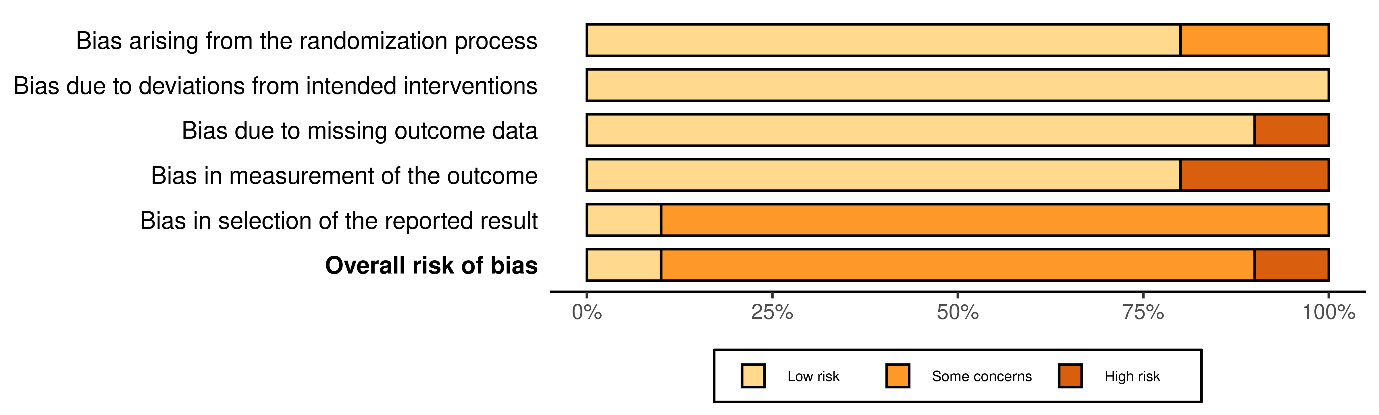
**

**Cognitive task descriptions**

**Table 3: Descriptions of the cognitive tasks as they were administered in each study**

| **Study** | **Cognitive function** | **Task** | **Task description** |
| --- | --- | --- | --- |
| Tomes & Katz, 1997 | Spatial imagery rotation | Paper Folding Task (Ekstrom et al., 1976) | Participants view a series of diagrams where a paper is folded, and hole is punched in specific location on the paper. At the end of the series, participants must select which of a series of unfolded papers reflects the hole-punch pattern from the diagram. There is no information in the article about how many trials, or how the PFT was scored. |
| Tomes & Katz, 1997 | Spatial imagery rotation | Card Rotation Task (Ekstrom et al., 1976) | Participants are shown one target shape. Then they are asked to select which out of a series of shapes have been rotated, and which have been mirrored. There is no information in the article about how many trials, or how the PFT was scored. |
| Jaschinski & Wentura, 2002 | Working memory capacity | Operation-Word Span (Turner & Engle, 1989) | “Participants were asked to perform a dual task. Their main task was to remember single words that were presented to them trial by trial. Simultaneously with the presentation of each word, participants had to judge whether arithmetical equations (e.g., 6*4+ 3= 27) were true or false. After a certain number of trials (e.g., five), participants were asked to recall the words. The index for working memory capacity is the sum of all remembered words during the test procedure” (Jaschinski & Wentura, 2002, pg. 226) |
| Parker et al., 2008 | Working memory capacity | Operation Span (Unsworth et al., 2005) | “ The OSPAN task consisted of 75 trials in which people solved mathematical equations while attempting to remember a sequence of random letters. This sequence continued until the end of each trial, at which point people had to choose, from a grid of 12 letters, which letters had been presented. There were three to seven letters per trial. To control for the possibility that people would concentrate only on remembering the letters and not solve the maths equations, maths accuracy had to remain above 85%; the computer issued a warning if accuracy dropped below this point. For each person we calculated an OSPAN score as the total number of letters correctly recalled on successfully completed trials.” (Parker et al., 2008, pg. 413) |
| Zhu et al., 2010 | Non-verbal intelligence | Raven’s Advanced Progressive Matrices (Raven et al., 1998) | “ These were multiple-choice tests of abstract reasoning. Participants were given 30 minutes to complete as many items as possible. In each test item, participants were asked to select from several alternatives the missing segment that would complete a larger pattern. The whole test had 48 items, including 12 easy items and 36 difficult items. Each item was presented in black ink against a white background. Items were arranged in the order of difficulty from the easiest to the most difficult… The total score on this test was one measure of intelligence used in the current study.” (Zhu et al., 2010, pg. 548) |
| Zhu et al., 2010 | Non-verbal intelligence | Weschler Adult Intelligence Scale - Performance (Gong, 1992) | Composite score of three different tests. “The performance section included picture completion (ability to quickly perceive visual details), symbol digit coding (visual-motor coordination and motor and mental speed), and block design (spatial perception, visual abstract processing, and problem solving)” (Zhu et al., 2010, pg. 548) |
| Zhu et al., 2010 | Verbal intelligence | Weschler Adult Intelligence Scale – Verbal (Gong, 1992) | Composite score of three different tests. “The verbal section included general knowledge (tapping general information acquired from one’s culture), similarities (abstract verbal reasoning), and digit span (attention and concentration).” (Zhu et al., 2010, pg. 548) |
| Zhu et al., 2010 | Working memory capacity | 2-back Task (Xue et al., 2004) | “Participants were presented with three series of characters (two series of Chinese characters and one series of Tibetan letters) sequentially and were asked to continuously judge whether the current character was related to the character presented two characters earlier (hence the name ‘‘2-back’’). There were three judgement tasks: semantic judgement (whether the characters were from the same semantic category, such as cabbage and radish) and phonological judgement (whether the characters rhymed) for the two series of Chinese characters, and morphemic judgement (whether two characters were the same) for Tibetan letters, which were unfamiliar and meaningless to participants in our study. Each judgement task consisted of four blocks and 10 trials in each block. tests….The average score (accuracy) of three tasks was used as the index of working memory in the current study.” (Zhu et al., 2010, pg. 549) |
| Zhu et al., 2010 | Short-term recall memory | Weschler Memory Scale – Recall (Gong, 1989) | “Participants were presented with 20 items to study for 90 seconds. These items were pictures of common objects, such as a boat or a cap. Afterwards, participants were asked to say out loud the items they just saw.” (Zhu et al., 2010, pg. 549). Score is amount of 20 items correctly recalled. |
| Zhu et al., 2010 | Short-term recognition memory | Weschler Memory Scale – Recognition (Gong, 1989) | “Participants were presented with eight items simultaneously for 30 seconds. Items included pictures of common objects and Chinese characters. Then participants were asked to pick out the 8 studied items from 28 items (including 8 studied and 20 unstudied similar items).” (Zhu et al., 2010, pg. 549). Score is amount of 8 items correctly recalled. |
| Zhu et al., 2010 | Visual perceptual discrimination ability | Motor-Free Visual Perception Test (Colarusso & Hammill, 2003) | “The participant was shown a line drawing and then asked to choose the matching drawing from a set of four presented on the following plate. Five categories of visual perception were measured: spatial relationship, visual closure, visual discrimination, visual memory, and figure ground. We used the standard score based on the score conversion table in Colarusso and Hammill (2003).” (Zhu et al., 2010, pg. 548) |
| Zhu et al., 2010 | Visual perceptual discrimination | Change Blindness Test (Rensink et al., 1997) | “Participants had to identify whether two pictures were the same or different and press a response key within 6 seconds. After 6 seconds without a response, a notice would be presented for 2 seconds asking for a response. The discrimination score (d?) on this test was used as the index of change blindness performance for the current study.” (Zhu et al., 2010, pg. 548) |
| Zhu et al., 2010 | Auditory perceptual discrimination | Tone Discrimination Test (Zatorre, 2003) | “Participants listened to a tone twice and were asked to choose the name of the tone. Seven piano tones were used, each presented three times in a random order. The number of correct responses in the formal test was used for analysis.” (Zhu et al., 2010, pg. 548) |
| Zhu et al., 2010 | Face recognition | Cambridge Face Memory Test (Duchaine & Nakayama, 2006) | “It had three blocks. First, participants saw a target face from the front, the left, and the right, and were then asked to identify the target face from three different faces. For the second block, participants saw six different faces, all presented at the same time for 20 seconds, and were then asked to identify one of these faces from three alternative choices (including one target face and two other foil faces each time). The third block was the same as the second block except that noise was added to the faces. For the first block, there were 6 target faces and 18 trials in total; for the second and the third block there were 6 target faces and 54 trials in total. The averaged accuracy rate for three blocks was used in this study.” (Zhu et al., 2010, pg. 549) |
| Zhu et al., 2010 | Facial expression recognition | Facial Expression Recognition Test (Wang & Markham, 1999) | “It assessed the ability of Chinese participants to judge facial expressions represented on Asian and Caucasian faces. Six basic emotions were included: happiness, surprise, anger, sadness, fear, and disgust. Participants selected from the six basic emotions to match it to each face. The total number of correct responses was used in this study.” (Zhu et al., 2010, pg. 549) |
| Calvillo, 2014 | Working memory capacity | Operation Span (Unsworth et al., 2005) | “Participants attempted to remember a series of letters separated by a math operation. The series of letters varied in length from three to seven, and at the end of a series, participants selected the letters that were presented, in the order in which they were presented, among a3× 4 matrix of letters.” (Calvillo, 2014, pg. 398) The total score of three blocks of the OSPAN was used was WMC score. |
| Calvillo, 2014 | Visual perceptual discrimination | Group Embedded Figures Test (Witkin et al., 1971) | “Participants searched for a specific figure embedded in a more complex figure in each item.” (Calvillo, 2014, pg. 398). The number of correct responses was the Visual perceptual discrimination score. |
| Nichols & Loftus, 2019 | Analytical reasoning | 3-item Cognitive Reflection Task (Frederick, 2005) | “…a three-item measure designed to gauge a subject’s willingness and ability to suppress an intuitive but incorrect answer to a problem and arrive at a correct answer.” (Nichols & Loftus, 2019, pg. 969). CRT scored with sum of correct responses and sum of intuitive responses as separate variables. |
| Farina & Greene, 2020 | Autobiographical memory specificity | Shortened Autobiographical Memory Specificity Task (de Decker et al., 2003) | “The test contained 10 emotional cue words: five positive (“happy”, “safe”, “interested”, “successful”, “surprised”) and five negative (“sad”, “evil”, “awkward”, “emotionally hurt”, “lonely”)... Participants had a maximum of 60 s to recall a specific memory in response to each cue. A specific memory was defined as a unique event that took place over the course of a single day and was more than 7 days old…Participants were provided with on-screen instructions for the duration of the test, stating that they should write down one specific memory for each cue. They were told that each memory should refer to one particular event lasting no more than a single day that occurred more than 1 week ago. Participants typed their responses into a text box below the cue word. The task automatically proceeded to the next cue after 60 s.” (Farina & Greene, 2020, pg. 930) Responses were coded as “specific” or “non-specific” memories by two independent coders. |
| Greene et al., 2020 | Working memory capacity | Operation Span (Unsworth et al., 2005) | “In each trial, participants are presented with an arithmetic equation (e.g. (4*5) – 5 = ?) and asked to indicate via mouse-click whether a proposed solution to the equation (e.g., 11) is true or false. At the end of each trial, participants are presented with a letter to remember. After a pre-determined number of trials, a 3 × 4 grid of letters is presented. Using the mouse, participants are asked to select the letters presented previously, in the correct order...In the experimental phrase of the task, participants complete 15 task sets, with three instances each of set size three, four, five, six and seven…The key outcome variable is the OSPAN score, calculated as the total number of letters recalled in the correct position across the whole task; the maximum achievable OSPAN score is 75.” (Greene et al., 2020, pg. 454) |
| Greene et al., 2020 | Verbal intelligence | Wordsum (Thorndike & Gallop, 1944) | “Participants are presented with a target word and asked to choose the word closest in meaning to the target from a list of 5 other words. For example, given the target word CLOISTERED, participants must choose the closest match from the words “miniature”, “bunched”, “arched”, “malady” and “secluded”. They may also select the option “don’t know”. (Greene et al., 2020, pg. 454). Wordsum score is number of correct responses out of 10. |
| Greene et al., 2020 | Analytical reasoning | 7-item Cognitive Reflection Task (Frederick, 2005, Toplack et al., 2014) | “…each item has an intuitive (but incorrect) answer and a correct answer that requires analytical reasoning. A sample question is “A bat and ball cost $1.10 in total. The bat costs $1.00 more than the ball. How much does the ball cost?” The intuitive answer to this question is 10c, whereas the correct answer is 5c.” (Greene et al., 2020; pg. 454). CRT score is number of correct scores out of 7. |
| Greene et al., 2021 | Verbal intelligence | Wordsum (Thorndike & Gallop, 1944) | “ Participants are presented with a target word and asked to choose the word closest in meaning to the target from a list of five other words. For example, given the target word CLOISTERED, participants must choose the closest match from the words “miniature”, “bunched”, “arched”, “malady”, and “secluded”. They may also select the option “don’t know”.” (Greene et al., 2021). Wordsum score is number of correct responses out of 10. |

**References**

Calvillo, D. P. (2014). Individual Differences in Susceptibility to Misinformation Effects and Hindsight Bias. *The Journal of General Psychology*, *141*(4), 393–407. <https://doi.org/10.1080/00221309.2014.954917>

Colarusso, R., & Hamill, D. (2003). *Motor-Free Visual Perception Test* (3rd ed.).

Daneman, M., & Carpenter, P. A. (1980). Individual differences in working memory and reading. *Journal of Verbal Learning and Verbal Behavior*, *19*(4), 450–466. <https://doi.org/10.1016/S0022-5371(80)90312-6>

de Decker, A., Hermans, D., Raes, F., & Eelen, P. (2003). Autobiographical Memory Specificity and Trauma in Inpatient Adolescents. *Journal of Clinical Child & Adolescent Psychology*, *32*(1), 22–31. <https://doi.org/10.1207/S15374424JCCP3201_03>

Duchaine, B., & Nakayama, K. (2006). The Cambridge Face Memory Test: Results for neurologically intact individuals and an investigation of its validity using inverted face stimuli and prosopagnosic participants. *Neuropsychologia*, *44*(4), 576–585. <https://doi.org/10.1016/j.neuropsychologia.2005.07.001>

Ekstrom, R., French, J., Harman, H., & Dermen, D. (1976). *Kit of Factor-Referenced Cognitive Tests*. ETS Educational Testing Service.

Farina, F. R., & Greene, C. M. (2020). Examining the effects of memory specificity and perceptual load on susceptibility to misleading information. *Applied Cognitive Psychology*, *34*(4), 928–938. <https://doi.org/10.1002/acp.3669>

Frederick, S. (2005). Cognitive Reflection and Decision Making. *Journal of Economic Perspectives*, *19*(4), 25–42. <https://doi.org/10.1257/089533005775196732>

Gong, Y., Jiang, D., Deng, J., Dai, Z., & Zhou, Q. (1992). *Wechsler Adult Intelligence Scale (WAIS-RC), Chinese Version.*

Gong, Y., Wang, D., & J Deng. (1989). *Handbook of Wechsler Memory Scale-Revised (WMS-RC), Chinese Version.* Changsha: Bulletin of Hunan Medical College.

Greene, C. M., Bradshaw, R., Huston, C., & Murphy, G. (2021). The medium and the message: Comparing the effectiveness of six methods of misinformation delivery in an eyewitness memory paradigm. *Journal of Experimental Psychology: Applied*. <https://doi.org/10.1037/xap0000364>

Greene, C. M., Maloney-Derham, R., & Mulligan, K. (2020). Effects of perceptual load on eyewitness memory are moderated by individual differences in cognitive ability. *Memory*, *28*(4), 450–460. <https://doi.org/10.1080/09658211.2020.1729811>

Jaschinski, U., & Wentura, D. (2002). Misleading postevent information and working memory capacity: An individual differences approach to eyewitness memory. *Applied Cognitive Psychology*, *16*(2), 223–231. <https://doi.org/10.1002/acp.783>

Kiat, J. E., Long, D., & Belli, R. F. (2018). Attentional responses on an auditory oddball predict false memory susceptibility. *Cognitive, Affective, & Behavioral Neuroscience*, *18*(5), 1000–1014. <https://doi.org/10.3758/s13415-018-0618-0>

McGuinness, L. A., & Higgins, J. P. T. (2020). *Risk-of-bias VISualization (robvis): An R package and Shiny web app for visualizing risk-of-bias assessments.* [R]. Res Syn Meth. <https://doi.org/10.1002/jrsm.1411>

Nichols, R. M., & Loftus, E. F. (2019). Who is susceptible in three false memory tasks? *Memory*, *27*(7), 962–984. <https://doi.org/10.1080/09658211.2019.1611862>

Parker, S., Garry, M., Engle, R. W., Harper, D. N., & Clifasefi, S. L. (2008). Psychotropic placebos reduce the misinformation effect by increasing monitoring at test. *Memory*, *16*(4), 410–419. <https://doi.org/10.1080/09658210801956922>

Powers, P. A., Andriks, J. L., & Loftus, E. F. (1979). Eyewitness accounts of females and males. *Journal of Applied Psychology*, *64*(3), 339–347. <https://doi.org/10.1037/0021-9010.64.3.339>

Raven, J., Raven, J. C., & Court, J. (1998). *Raven Manual: Section 4, Advanced Progressive Matrices, 1998 Edition.* Oxford Psychologists Press Ltd.

Rensink, R. A., O’Regan, J. K., & Clark, J. J. (1997). To See or not to See: The Need for Attention to Perceive Changes in Scenes. *Psychological Science*, *8*(5), 368–373. <https://doi.org/10.1111/j.1467-9280.1997.tb00427.x>

Sterne, J. A. C., Savović, J., Page, M. J., Elbers, R. G., Blencowe, N. S., Boutron, I., Cates, C. J., Cheng, H.-Y., Corbett, M. S., Eldridge, S. M., Emberson, J. R., Hernán, M. A., Hopewell, S., Hróbjartsson, A., Junqueira, D. R., Jüni, P., Kirkham, J. J., Lasserson, T., Li, T., … Higgins, J. P. T. (2019). RoB 2: A revised tool for assessing risk of bias in randomised trials. *BMJ*, *366*, l4898. <https://doi.org/10.1136/bmj.l4898>

Thorndike, R. L., & Gallup, G. H. (1944). Verbal intelligence of the American adult. *Journal of General Psychology*, *30*, 75–85. <https://doi.org/10.1080/00221309.1943.10544458>

Tomes, J. L., & Katz, A. N. (1997). Habitual Susceptibility to Misinformation and Individual Differences in Eyewitness Memory. *Applied Cognitive Psychology*, *11*(3), 233–251. [https://doi.org/10.1002/(SICI)1099-0720(199706)11:3<233::AID-ACP447>3.0.CO;2-V](https://doi.org/10.1002/(SICI)1099-0720(199706)11:3%3c233::AID-ACP447%3e3.0.CO;2-V)

Toplak, M. E., West, R. F., & Stanovich, K. E. (2014). Assessing miserly information processing: An expansion of the Cognitive Reflection Test. *Thinking & Reasoning*, *20*(2), 147–168. <https://doi.org/10.1080/13546783.2013.844729>

Turner, M. L., & Engle, R. W. (1989). Is working memory capacity task dependent? *Journal of Memory and Language*, *28*(2), 127–154. <https://doi.org/10.1016/0749-596X(89)90040-5>

Unsworth, N., Heitz, R. P., Schrock, J. C., & Engle, R. W. (2005). An automated version of the operation span task. *Behavior Research Methods*, *37*(3), 498–505. <https://doi.org/10.3758/BF03192720>

Wang, L., & Markham, R. (1999). The Development of a Series of Photographs of Chinese Facial Expressions of Emotion. *Journal of Cross-Cultural Psychology*, *30*(4), 397–410. <https://doi.org/10.1177/0022022199030004001>

Witkin, H., Oltman, P., Raskin, E., & Karp, S. (1971). *A manual for the group embedded figures test.* Consulting Psychologists Press.

Xue, G., Dong, Q., Jin, Z., & Chen, C. (2004). Mapping of verbal working memory in nonfluent Chinese–English bilinguals with functional MRI. *NeuroImage*, *22*(1), 1–10. <https://doi.org/10.1016/j.neuroimage.2004.01.013>

Zatorre, R. J. (2003). Absolute pitch: A model for understanding the influence of genes and development on neural and cognitive function. *Nature Neuroscience*, *6*(7), Article 7. <https://doi.org/10.1038/nn1085>

Zhu, B., Chen, C., Loftus, E. F., Lin, C., He, Q., Chen, C., Li, H., Xue, G., Lu, Z., & Dong, Q. (2010). Individual differences in false memory from misinformation: Cognitive factors. *Memory*, *18*(5), 543–555
